# A Role for the RNA Polymerase Gene Specificity Factor σ^54^ in the Uniform Colony Growth of Uropathogenic Escherichia coli

**DOI:** 10.1128/jb.00031-22

**Published:** 2022-03-31

**Authors:** Amy Switzer, Lynn Burchell, Panagiotis Mitsidis, Teresa Thurston, Sivaramesh Wigneshweraraj

**Affiliations:** a MRC Centre for Molecular Bacteriology and Infection, Imperial College London, London, United Kingdom; Duchossois Family Institute

**Keywords:** *Escherichia coli*, RNA polymerase, sigma 54, sigma factors, UPEC, microcolonies, transcription

## Abstract

The canonical function of a bacterial sigma (σ) factor is to determine the gene specificity of the RNA polymerase (RNAP). In several diverse bacterial species, the σ^54^ factor uniquely confers distinct functional and regulatory properties on the RNAP. A hallmark feature of the σ^54^-RNAP is the obligatory requirement for an activator ATPase to allow transcription initiation. Different activator ATPases couple diverse environmental cues to the σ^54^-RNAP to mediate adaptive changes in gene expression. Hence, the genes that rely upon σ^54^ for their transcription have a wide range of different functions suggesting that the repertoire of functions performed by genes, directly or indirectly affected by σ^54^, is not yet exhaustive. By comparing the growth patterns of prototypical enteropathogenic, uropathogenic, and nonpathogenic Escherichia coli strains devoid of σ^54^, we uncovered that the absence of σ^54^ results in two differently sized colonies that appear at different times specifically in the uropathogenic E. coli (UPEC) strain. Notably, UPEC bacteria devoid of individual activator ATPases of the σ^54^-RNAP do not phenocopy the σ^54^ mutant strain. Thus, it seems that σ^54^’s role as a determinant of uniform colony appearance in UPEC bacteria represents a putative non-canonical function of σ^54^ in regulating genetic information flow.

**IMPORTANCE** RNA synthesis is the first step of gene expression. The multisubunit RNA polymerase (RNAP) is the central enzyme responsible for RNA synthesis in bacteria. The dissociable sigma (σ) factor subunit directs the RNAP to different sets of genes to allow their expression in response to various cellular needs. Of the seven σ factors in Escherichia coli and related bacteria, σ^54^ exists in a class of its own. This study has uncovered that σ^54^ is a determinant of the uniform growth of uropathogenic E. coli on solid media. This finding suggests a role for this σ^54^ in gene regulation that extends beyond its known function as an RNAP gene specificity factor.

## INTRODUCTION

Central to bacterial gene expression is transcription, which is the step where RNA synthesis occurs. Transcription in bacteria is catalyzed by the multisubunit enzyme, RNA polymerase (RNAP). For promoter-specific initiation of transcription, the catalytic complex of the RNAP (α_2_ββ'ω) must associate with one of the many different sigma (σ) factor subunits. The primary function of σ factors is to direct the RNAP to the promoters of specific sets of genes. Many bacterial genomes contain multiple σ factors, which reversibly and competitively bind to the RNAP to execute and coordinate specific transcription programs to regulate cellular processes under a particular growth condition. As such, σ factors can be considered master regulatory factors governing bacterial gene expression. Based on functional and structural criteria, bacterial σ factors are grouped into two distinct classes (1). In Escherichia coli, six of the seven σ factors belong to the σ^70^ class, named after the prototypical housekeeping σ factor, σ^70^. The σ^54^ factor, which is historically associated with executing transcriptional programs that allow bacteria to cope with conditions of nitrogen adversity (2) and found in genomes of many phylogenetically diverse bacteria, exists in a class of its own (3, 4).

The σ^70^ and σ^54^ classes of σ factors confer distinct regulatory properties upon the RNAP. The σ^70^ class of σ factors (referred to as σ^70^ for simplicity) direct the RNAP to promoters with conserved sequences centered at −35 and −10 bp (bp) upstream from the transcription start site (+1 site). In contrast, σ^54^ directs its RNAP to promoters characterized by conserved sequences located at −24 and −12 bp upstream of the +1 site. The initial binding of the RNAP to the promoter results in a closed promoter complex (RPc). Regulation of transcription at most σ^70^-dependent promoters often occurs to either stimulate or antagonize RPc formation. The RPc at σ^70^-dependent promoters is usually short-lived and either dissociates or spontaneously isomerizes to the transcriptionally proficient open promoter complex (RPo). In the RPo, the promoter DNA strands are locally melted, such that the single-stranded +1 site is positioned in the catalytic cleft of the RNAP to allow the initiation of RNA synthesis. The RPc at σ^54^-dependent promoters rests in a ‘ready-to-respond’ state for RPo formation. Conversion of the RPc to RPo at σ^54^-dependent promoters requires a specialized activator ATPase. Different activator ATPases sense and couple diverse environmental and intracellular signals to activate specific sets of σ^54^-dependent promoters. The activator ATPase binds to cognate DNA sites, called enhancers, located ∼100 to 150 bp frequently upstream, but sometimes downstream, of σ^54^-dependent promoters. The interaction between the enhancer-bound activator ATPase and the RPc occurs via a DNA-looping event and results in ATP hydrolysis-dependent conformational rearrangements in the σ^54^, the catalytic subunits of the RNAP, and the promoter DNA to allow RPo formation.

The reliance on different specialized activator ATPases allows the σ^54^-containing RNAP (σ^54^-RNAP) to rapidly activate specific transcription programs to respond to a particular growth condition. As such, the products of genes that depend on σ^54^ have a wide range of different functions in E. coli and several related bacteria, but no obvious single theme appears in the repertoire of functions performed by their products (5). Many animal and plant-pathogenic bacteria rely on the σ^54^ for transcription of genes linked to virulence or virulence-associated processes (reviewed in reference (6)). E. coli is a commensal inhabitant of the mammalian gastrointestinal tract, but E. coli lineages have acquired specific virulence characteristics, which confer them the capacity to adapt and thrive in specific host niches, causing significant morbidity and mortality as human pathogens. Although pathogenic E. coli can cause intestinal/enteric and extraintestinal infections, both with the potential to lead to serious systemic disease, most of our knowledge of the involvement of σ^54^ in E. coli pathogenesis is limited to studies on the intestinal pathogen, enterohemorrhagic E. coli (EHEC). To establish in the host, EHEC bacteria must overcome the acidic gastrointestinal environment and the σ^54^ is involved in the cascade of processes associated with conferring acid resistance and attachment to intestinal cells for competitive host colonization (6–9).

Uropathogenic E. coli (UPEC) are members of extraintestinal pathogenic E. coli and are the major causative agent of urinary tract infections (UTIs) worldwide. UTIs are among the most common bacterial infections in humans in community and nosocomial settings. UPEC infections range in severity and are a health care problem compounded by the emergence of antibiotic-resistant UPEC isolates (reviewed in references (10, 11)). UPEC bacteria normally reside in the gastrointestinal tract but cause disease when they gain entry to the urinary tract leading to cystitis, the most common form of UTI. UPEC bacteria also invade the cytoplasm of uroepithelial cells, replicate and form intracellular bacterial communities (IBCs). Although the host immune system may remove some of the IBCs and excrete them with the urine, the remaining bacteria can exist as a biofilm resistant to host immune responses and antibiotic treatment. Some UPEC bacteria can escape from the biofilm and disseminate into the bladder lumen causing recurrent episodes of cystitis. Some can ascend into the kidneys causing pyelonephritis, and some may also spread from the urinary tract to the bloodstream causing bacteremia. Therefore, UPEC bacteria need to efficiently adjust their transcriptional programs to adapt and survive in diverse host environments, resist assaults from the host’s immune system, and tolerate antibiotic therapy (12–14). Although previous studies have shown markers of nitrogen limitation to be upregulated in UPEC isolated from the urine of both UPEC-infected mice and women with UTIs, suggesting σ^54^ is likely required in the coordination of the nitrogen stress response under these conditions (14, 15), little, if any, information exists on the role of σ^54^ in UPEC physiology and pathogenesis. We report a hitherto unexpected role for σ^54^ in the UPEC strain CFT073, which was originally isolated from the blood and urine of a woman suffering from pyelonephritis (16).

## RESULTS

### The growth properties of different E. coli strains lacking σ^54^.

**FIG 1:**
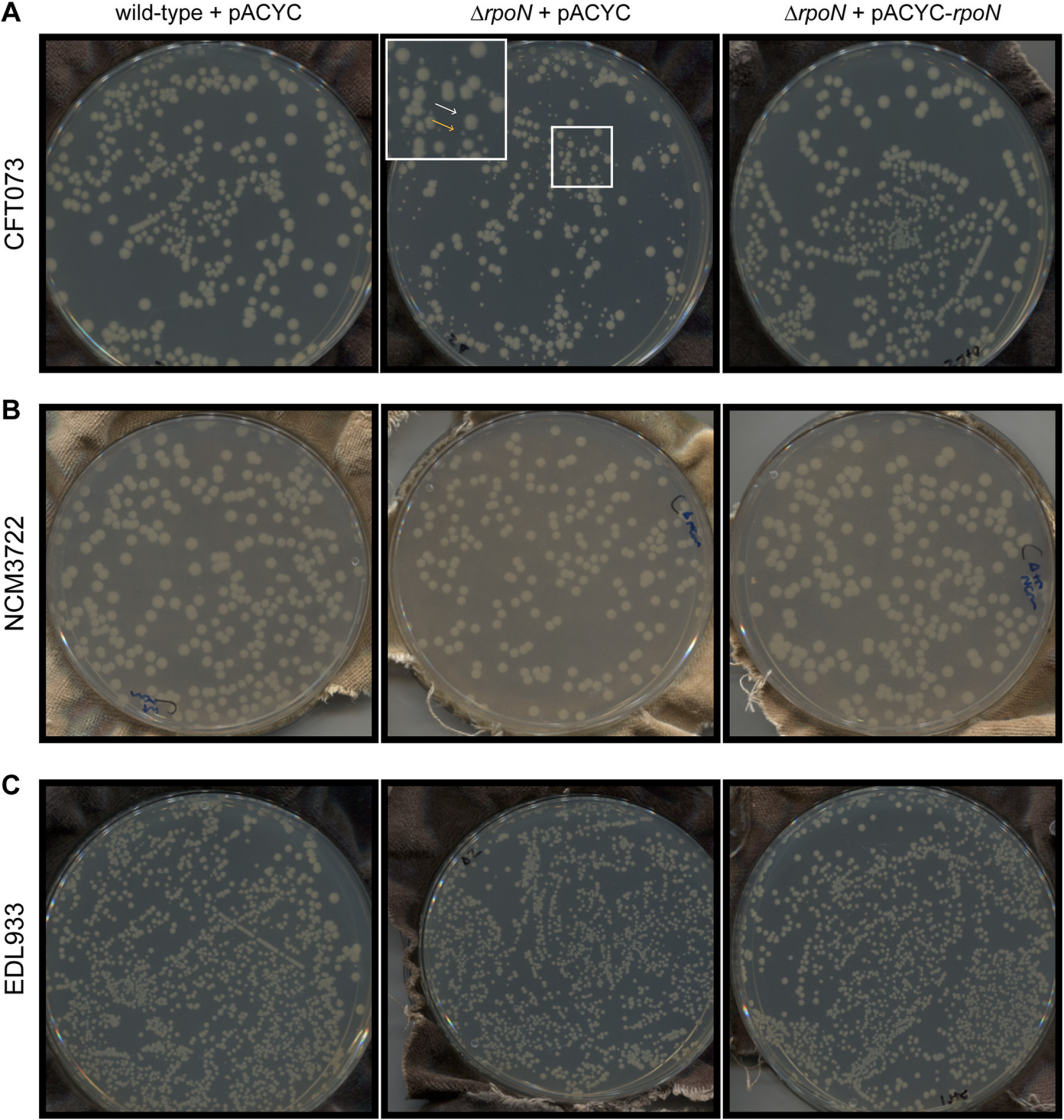
The growth properties of strains of E. coli lacking σ^54^. Images of colonies on LB agar plates incubated at 33°C for 48 h containing wild-type (left), Δ*rpoN* (middle) or Δ*rpoN* + *rpoN* (right) of E. coli strains (A) CFT073, (B) NCM3722, and (C) EDL933. The two differently sized colonies seen on plates containing the Δ*rpoN* CFT073 strain are indicated with white (big colony) and orange (small colony) arrows in the inset in (A).

We generated a Δ*rpoN* CFT073 strain and, intriguingly, when plated onto LB agar plates, we consistently observed two differently sized colonies following 48 h of incubation at 37°C (Fig. 1A). In contrast, the wild-type CFT073 colonies were more uniform in size. The difference in the colony size observed with the Δ*rpoN* CFT073 strain was reversible when *rpoN* was exogenously supplied via a low copy number plasmid from its native promoter (pACYC-*rpoN*). Although the protein sequence of σ^54^ in CFT073 differed by two amino acids in NCM3722 (a prototypic wild-type K-12 E. coli strain related to strain MG1655; (17)) but was identical to EDL933 (an EHEC strain), we did not detect any obvious difference in colony size when the Δ*rpoN* NCM3722 or the Δ*rpoN* EDL933 strains were plated onto LB agar plates. In addition, the mutant colonies resembled those formed by the respective wild-type strains (Fig. 1B and C, respectively).

**FIG 2:**
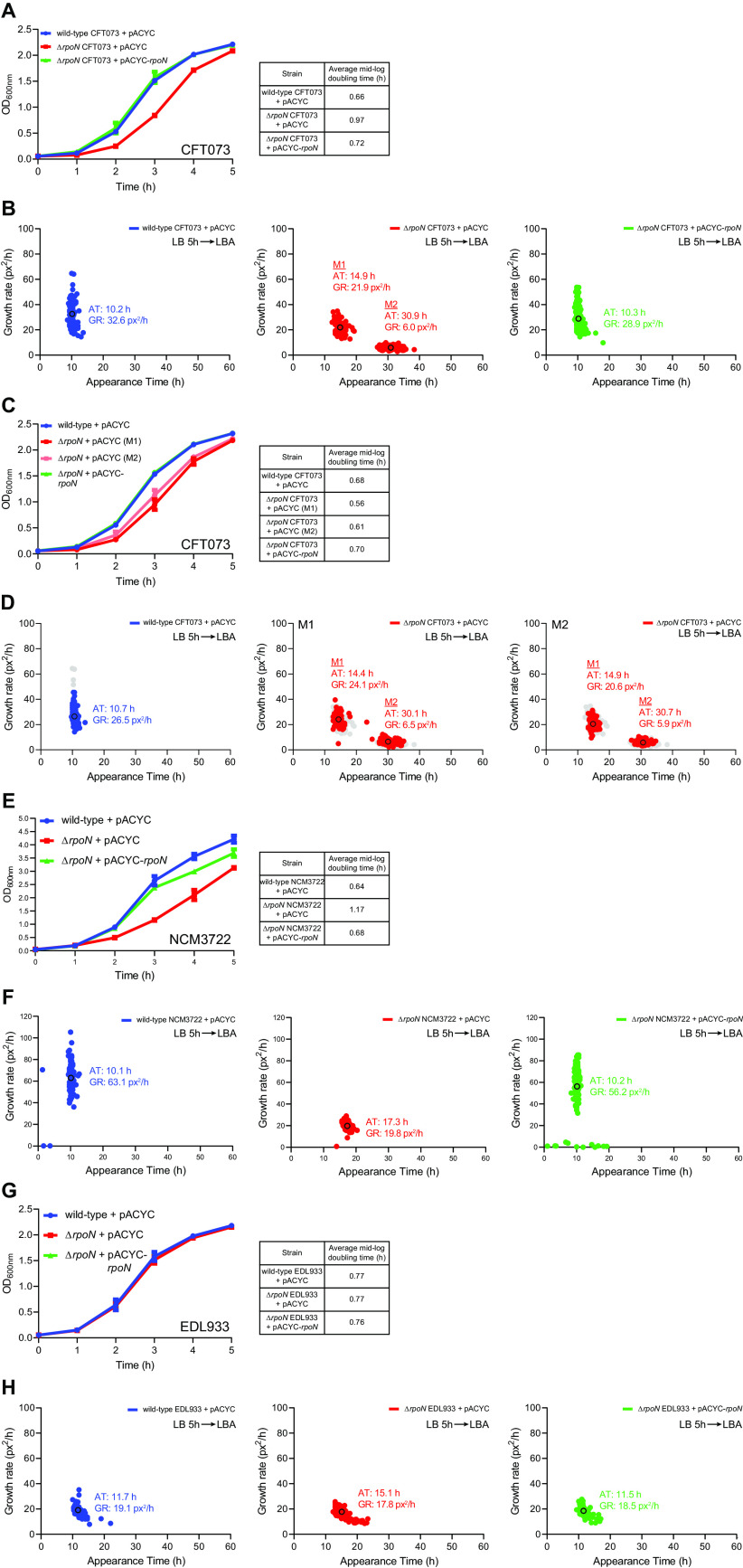
The growth properties of different strains of E. coli lacking σ^54^. (A) The growth curves of wild-type (blue), Δ*rpoN* (red), and Δ*rpoN* + *rpoN* (green) CFT073 strains grown in LB liquid medium for 5 h. (B) Scanlag analysis of colony appearance time (AT; h) and growth rate (GR) in pixels^2^/h (px^2^/h) for CFT073 strains grown in LB liquid medium for 5 h of wild-type (left), Δ*rpoN* (red), and Δ*rpoN* + *rpoN* (green) and plated onto LB agar (LBA) plates. Black circles represent average population growth rate and appearance time with mean average values indicated. Liquid to solid growth conditions is indicated in the top right. (C) The growth curves in LB liquid medium from colonies picked from (B) and color-coded as indicated. The average doubling time for each colony type is indicated in table (D) Scanlag analysis as in (B) for bacteria plated from (C). Shown in gray is data from (B) for reference. (E) The growth curves of wild-type (blue), Δ*rpoN* (red), and Δ*rpoN* + *rpoN* (green) NCM3722 strains grown in LB liquid medium for 5 h. (F) Scanlag analysis of colony appearance time as in (B) for NCM3722 strains. (G) The growth curves of wild-type (blue), Δ*rpoN* (*red*), and Δ*rpoN* + *rpoN* (green) EDL933 strains grown in LB liquid medium for 5 h. (H) Scanlag analysis of colony appearance time as in (B) for EDL933 strains grown in LB liquid medium for 5 h. Where indicated, the error bars represent standard deviation (*n* = 3).

To better understand how σ^54^ contributes to the growth dynamics of the three different E. coli strains on LB agar plates, particularly the CFT073 strain, we used a method called ScanLag (18). This involves putting the inoculated LB agar plates into a conventional office scanner, which was placed in a 33°C incubator and periodically (in this case, every 20 min) activating the scanner to measure the appearance time (in hours; h) of individual colonies and their apparent growth rate. The growth rate was expressed in pixels^2^/h (px^2^/h), which represented the area the colony increased hourly as measured in pixels^2^ from the image (where a diameter of 1-pixel measures approximately 0.1 mm) and was calculated during the first 6 h after the initial detectable appearance of colonies. To inoculate the bacteria onto LB agar plates, we subcultured LB liquid cultures of the wild-type, Δ*rpoN* and Δ*rpoN* + pACYC-*rpoN* bacteria that were grown overnight into fresh LB liquid medium and grew the bacteria for 5 h at 37°C. At this stage, the bacteria were in the early stationary-phase of growth (see below), and we plated 100 μL of each culture diluted by approximately 10^−5^ to 10^−6^ for the ScanLag experiment. For the Δ*rpoN* CFT073 strain, we initially used a ‘big’ colony (see Fig. 1A) for the ScanLag experiments. The wild-type CFT073 strain had a doubling time of ∼0.66 h in LB liquid medium (Fig. 2A), and its colonies appeared ∼10 h after incubation and had an apparent growth rate of ∼33 px^2^/h (Fig. 2B). The ‘big’ colony of the Δ*rpoN* CFT073 strain had a slightly longer doubling time in LB liquid medium compared to the wild-type strain (Fig. 2A). Notably, the appearance time of about half the proportion of the Δ*rpoN* CFT073 colonies (referred to as M1) was delayed by ∼5 h compared to the wild-type strain and had an apparent growth rate of ∼22 px^2^/h, while the appearance time of the remaining proportion of the Δ*rpoN* CFT073 colonies (referred to as M2) was delayed by ∼20 h compared to the wild-type strain and had an apparent growth rate of ∼6 px^2^/h (Fig. 2B). Here after, we refer to the appearance pattern of the Δ*rpoN* CFT073 colonies as heterogeneous. The colonies of the Δ*rpoN* CFT073 bacteria containing plasmid pACYC-*rpoN*, which exogenously produces σ^54^ from its native promoter, as expected, neither displayed a growth defect in LB liquid medium (Fig. 2A) nor the heterogeneous colony appearance pattern (Fig. 2B). We then picked the M1 and M2 colonies from the LB agar plate, created an overnight inoculum in LB liquid media, and compared their growth properties in fresh LB liquid medium and ScanLag experiments. As shown in Fig. 2C, the doubling time of the bacteria from the M1 and M2 colonies did not differ, suggesting that the slow apparent growth rate of the M2 colony on LB solid medium is reversible in LB liquid media. Consistent with this view, in ScanLag experiments, bacteria that were grown from M1 and M2 colonies displayed an indistinguishable heterogeneous appearance pattern on LB agar plates (Fig. 2D). Although the doubling time of the Δ*rpoN* NCM3722 strain was also compromised in LB liquid medium (like the Δ*rpoN* CFT073 strain) (Fig. 2E), the Δ*rpoN* NCM3722 strain, consistent with results in Fig. 1B, did not display a heterogeneous appearance pattern on LB agar plates (Fig. 2F). However, the appearance time of Δ*rpoN* NCM3722 colonies on LB agar plates was delayed by ∼7 h (apparent growth rate ∼20 px^2^/h) compared to the wild-type NCM3722 colonies (apparent growth rate ∼63 px^2^/h) (Fig. 2F). The Δ*rpoN* EDL933 strain neither displayed any growth defect in LB liquid medium (Fig. 2G) nor the heterogeneous appearance pattern on LB agar plates (Fig. 2H). However, the appearance time of Δ*rpoN* EDL933 colonies on LB agar plates was delayed by ∼5 h (apparent growth rate ∼18 px^2^/h) compared to the wild-type EDL933 colonies (apparent growth rate ∼19 px^2^/h) (Fig. 2G). As with the Δ*rpoN* CFT073 strain, the presence of plasmid pACYC-*rpoN* reverted the LB liquid growth defect (where applicable) and returned colony appearance time of the Δ*rpoN* NCM3722 and Δ*rpoN* EDL933 strains to their respective wild-type levels. The absence of σ^54^ in E. coli strains NCM3722, EDL933, and CFT073 resulted in a notably delayed appearance of colonies on LB agar plates despite only having a modest adverse effect on growth in LB liquid media. In the case of the E. coli strain CFT073, the absence of σ^54^ significantly delayed the appearance time of a subpopulation of bacteria on LB agar plates (i.e., the M2 colonies), resulting in a heterogeneous colony appearance pattern. The delayed appearance of this subpopulation of Δ*rpoN* CFT073 colonies was a property specific to the CFT073 strain because the heterogeneous colony appearance pattern was not apparent with the NCM3722 and EDL933 strains. In conclusion, it seems that σ^54^ determines uniform colony growth of E. coli strain CFT073.

### Heterogeneous colony appearance is an inherent property of Δ*rpoN* CFT073 bacteria.

**FIG 3:**
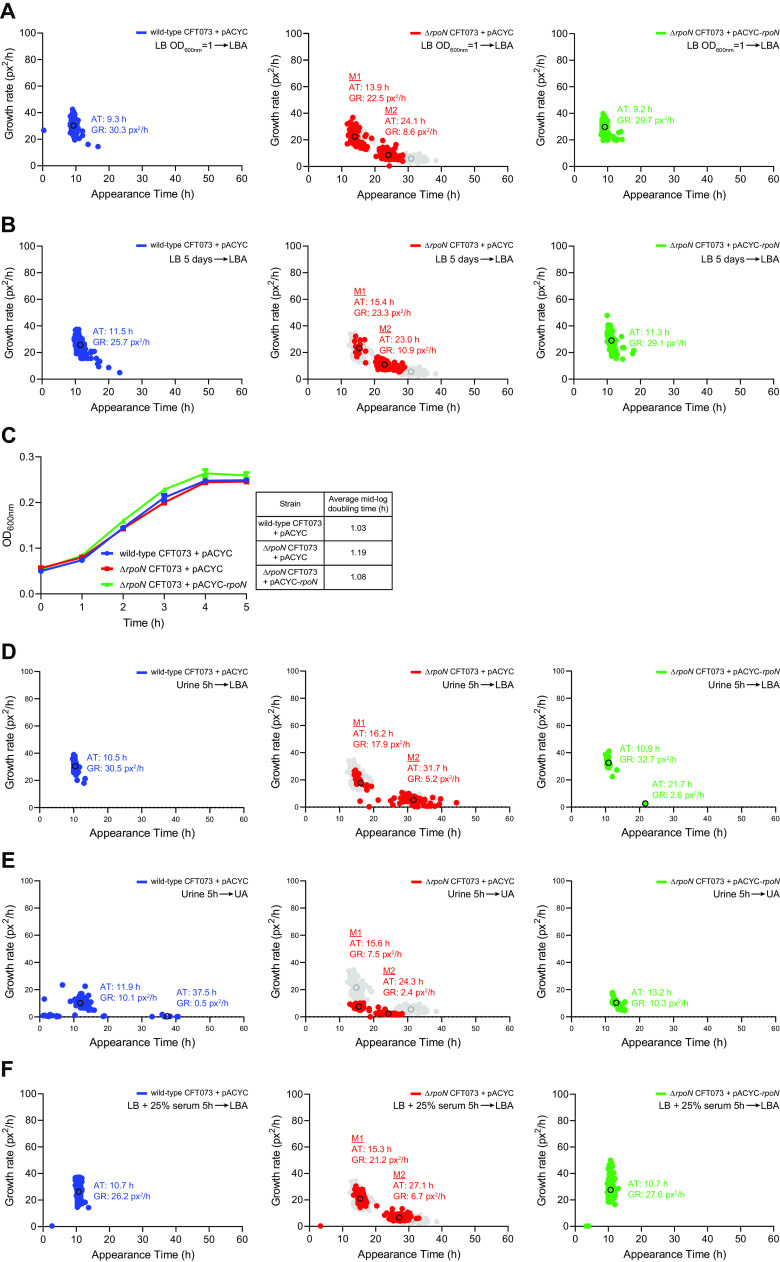
Heterogeneous colony appearance is an inherent property of Δ*rpoN* CFT073 bacteria. (A) Scanlag analysis (as in Fig. 2) of CFT073 bacteria grown in LB liquid medium to mid-exponential-phase before plating on LB agar (LBA) plates. Comparison of the M1 and M2 colonies from bacteria grown in LB liquid medium for 5 h before plating (gray). (B) As in (A) but with bacteria grown for 5 days in LB liquid media. (C) Growth curves of wild-type (blue), Δ*rpoN* (red), and Δ*rpoN* + *rpoN* (green) CFT073 strains grown in healthy female urine (HFU) for 5 h. (D) As in (A) but with bacteria from (C). (E) As in (D) but bacteria were plated onto agar plates containing 50% (vol/vol) HFU (UA). (F) As in (A) but bacteria were grown in LB liquid medium with 25% (vol/vol) human serum for 5 h before plating. Where indicated, the error bars represent standard deviation (*n* = 3).

We wanted to determine how the appearance time of colonies M1 and M2 of the Δ*rpoN* CFT073 strain are affected by the growth stage and culture conditions of the bacteria used for the ScanLag experiments. The colony appearance time and pattern of stationary-phase bacteria on LB agar plates (Fig. 2B) served as a reference condition (here after shown in gray in all applicable figures). As shown in Fig. 3A, when exponentially growing bacteria were used for the ScanLag experiment, both M1 and M2 colonies were present. However, we note that the M2 colonies of exponentially growing bacteria appeared sooner (by ∼5 h) than the M2 colonies of early stationary-phase bacteria. Similarly, when bacteria from a 5-day-old culture were used, the M2 colonies were still present but they appeared sooner (by ∼8 h) than the M2 colonies of early stationary-phase bacteria (Fig. 3B). We next wanted to determine how bacteria that were grown in healthy female urine (HFU) affected the appearance time of colonies M1 and M2 on LB agar plates. As shown in Fig. 3C, the doubling time of the Δ*rpoN* CFT073 strain in HFU did not substantially differ from that of the wild-type CFT073 strain. Although, unsurprisingly, the doubling time of all strains was markedly slower in HFU than in LB liquid medium (Fig. 3C and Fig. 2A, respectively). In the ScanLag experiments, the heterogeneous colony appearance pattern of Δ*rpoN* CFT073 colonies were evident, but the apparent growth rates of both M1 and M2 colonies were reduced compared to those of Δ*rpoN* CFT073 grown in LB (Fig. 3D and Fig. 2B, respectively). We also conducted ScanLag experiments with HFU-grown bacteria plated onto agar plates containing 50% (vol/vol) of HFU. In this case, the apparent growth rates of colonies of wild-type, Δ*rpoN* and Δ*rpoN* containing plasmid pACYC-*rpoN* CFT073 strains were reduced (Fig. 3E). Although the colonies of the Δ*rpoN* strain appeared in a heterogeneous fashion, we note a marked change in the timing of appearance of the M2 colonies, which appeared sooner (by ∼5 h) than the M2 colonies of the HFU-grown Δ*rpoN* bacteria plated onto normal LB agar plates (Fig. 3D and E). We also note the presence of a small population of wild-type colonies that appear ∼37 to 40 h after incubation, but like in other conditions, most of the wild-type colonies appeared ∼10 h after incubation. Finally, as the CFT073 strain was also isolated from blood (16), we wanted to determine how bacteria grown in LB liquid medium supplemented with 25% (vol/vol) human serum affected the appearance time of colonies M1 and M2 on LB agar plates. As shown in Fig. 3E, we note that the M2 colonies of bacteria grown in LB liquid medium with 25% (vol/vol) human serum appeared ∼4 h earlier than the M2 colonies of early stationary-phase bacteria. Under all conditions tested, as expected, the appearance time of colonies of the Δ*rpoN* CFT073 bacteria containing plasmid pACYC-*rpoN* resembled that of the wild-type strain. Overall, it seemed that the heterogeneous appearance of colonies was an inherent property of the CFT073 strain when *rpoN* was absent and that the slower-growing subpopulation of colonies (i.e., the M2 colonies) appear to be more sensitive to the growth environment and conditions than the faster-growing colonies (i.e., the M1 colonies). In conclusion, the results underscore that σ^54^ is a determinant for uniform colony growth of E. coli CFT073.

### Consequences of heterogeneous colony growth on virulence and fitness of CFT073 bacteria.

**FIG 4:**
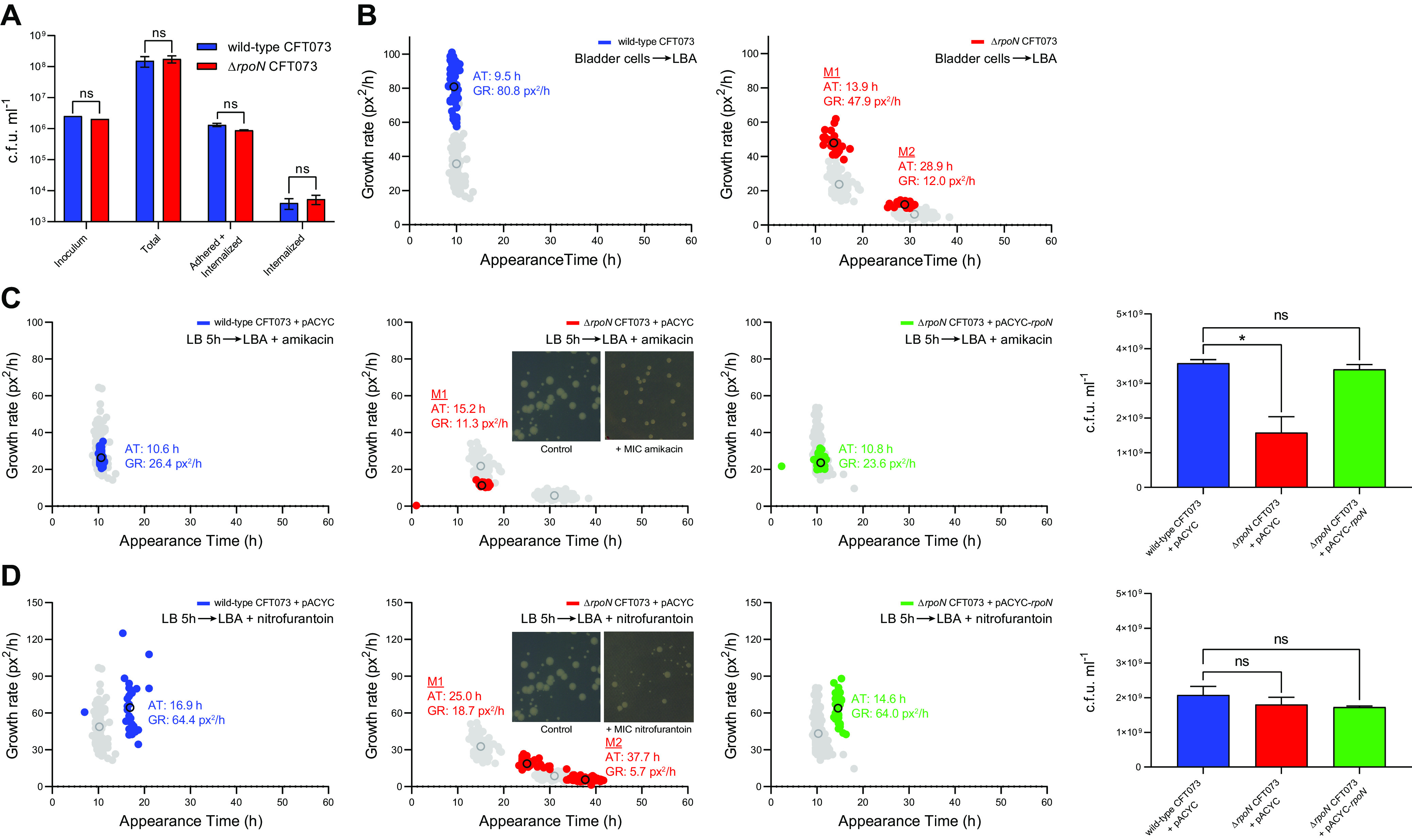
Consequences of heterogeneous colony growth on virulence and fitness of CFT073 bacteria. (A) The viability of wild-type (blue) and Δ*rpoN* (red) CFT073 bacteria as measured by CFU of added, total, adhered and internalized, and internalized only bacteria (see the text for details). (B) Scanlag analysis shows colony appearance time (AT; h) and growth rate in pixels^2^/h (GR; px^2^/h) for internalized only wild-type (blue, middle) and Δ*rpoN* (red, right) CFT073 strains plated onto LB agar (LBA) plates. Black circles represent average population growth rate and appearance time with mean average values indicated. Liquid to solid growth conditions are indicated in the top right. Shown in gray for comparison are the colonies from bacteria grown in LB liquid medium for 5 h before plating on LBA. (C) Scanlag analysis of wild-type (blue), Δ*rpoN* (red), and Δ*rpoN* + *rpoN* (green) CFT073 bacteria following growth in LB liquid medium for 5 h followed by plating on LBA plates with MIC of amikacin. Shown in gray for comparison are the colonies from bacteria grown in LB liquid medium for 5 h before plating on LB agar containing no antibiotics. (D) As in (C), but bacteria were plated on LBA plates containing MIC of nitrofurantoin. The inset images show representative colonies for comparison (see the text). In (C) and (D), the bar graphs indicate viability as measured by CFU of wild-type (blue), Δ*rpoN* (red), and Δ*rpoN* + *rpoN* (green) CFT073 bacteria plated on LBA plates with MIC of amikacin or nitrofurantoin, respectively. Statistical significance was calculated using one-way ANOVA with a probability (*P*) value of <0.05 deemed statistically significant (*, *P* < 0.05; ns, not significant). Error bars represent standard deviation (*n* = 3).

As σ^54^ is highly conserved and known to play a role in virulence in diverse bacterial species (6), we investigated how the absence of σ^54^, which is a determinant of the uniform colony, i.e., nonplanktonic, growth, affects host cell internalization and survival. To do this, bacteria were grown overnight at 37°C shaking in RPMI liquid medium supplemented with 10% (vol/vol) fetal calf serum (FCS) and added to bladder epithelial cell line 5637 at a multiplicity of infection of 50 for 2 h at 37°C. CFU was determined for the inoculum (bacteria before adding to bladder epithelial cells), total (all bacteria extracted from the wells after 2 h of incubation with bladder epithelial cells), adhered, and internalized (all bacteria that are exclusively adhered and/or internalized into bladder epithelial cells), and internalized only bacteria obtained after 1 h of treatment with gentamicin to kill extracellular bacteria. As shown in Fig. 4A, the numbers of total, adhered and internalized, and internalized only bacteria recovered from coculture with the bladder epithelial cells was similar for both wild-type and Δ*rpoN* CFT073 bacteria, suggesting that the absence of σ^54^ does not have any detectable adverse effect on the attachment, invasion, and survival of CFT073 bacteria in bladder epithelial cells under our conditions. Notably, when we conducted ScanLag analysis with internalized bacteria extracted from the bladder epithelial cells, the Δ*rpoN* CFT073 bacteria displayed the characteristic heterogeneous colony appearance pattern (Fig. 4B) (as also observed with bacteria grown in LB liquid broth, HFU or LB liquid broth supplemented with serum shown in Fig. 3), consistent with the view that heterogeneous colony appearance is an inherent property of Δ*rpoN* CFT073 bacteria. We note that both wild-type and Δ*rpoN* CFT073 bacterial colonies of internalized bacteria had markedly higher growth rates than the colonies of LB or HFU grown bacteria (Fig. 4B).

As it is widely accepted that growth heterogeneity can confer considerable fitness benefits to a bacterial population, we considered how the heterogeneous appearance of colonies of Δ*rpoN* CFT073 bacteria affected fitness under growth-restrictive conditions. Because UPEC bacteria are frequently exposed to antibiotics, we investigated the sensitivity of M1 and M2 colonies of Δ*rpoN* CFT073 bacteria to MICs of amikacin and nitrofurantoin – two frequently used antibiotics to treat UTIs caused by UPEC. We did this by conducting ScanLag analysis using LB agar plates containing MICs of amikacin or nitrofurantoin. As shown in Fig. 4C, we failed to detect any M2 colonies on LB agar plates containing MIC of amikacin, although the appearance time of M1 colonies was like that of bacteria on LB agar plates without any antibiotics, they grew considerably slower on antibiotic-containing plates. Consistent with this observation, the viability of Δ*rpoN* CFT073 bacteria was significantly reduced compared with wild-type CFT073 bacteria on LB agar plates with MIC of amikacin, suggesting that, rather than reverting to an M1 phenotype, the M2 population is unable to grow in the presence of MIC levels of amikacin. In contrast, we detected the characteristic heterogeneous growth property of the Δ*rpoN* CFT073 bacteria on LB agar plates containing nitrofurantoin (Fig. 4D). However, the appearance time and apparent growth rate of both M1 and M2 colonies were markedly compromised compared to their growth on LB agar plates without any antibiotics, to such a degree that the M2 colonies were barely detectable (Fig. 4D). However, the overall viability of Δ*rpoN* CFT073 bacteria was comparable to wild-type CFT073 bacteria. We noted that the appearance time and apparent growth rate of wild-type CFT073 bacteria on nitrofurantoin-containing LB agar plates were also compromised but not to the degree observed with the Δ*rpoN* CFT073 bacteria. Collectively, it seems that uniform colony growth, which requires *rpoN*, is likely to confer fitness advantages to the E. coli CFT073 strain under growth-restrictive conditions.

### Comparative analysis of the transcriptomes of wild-type and Δ*rpoN* CFT073 strains.

**FIG 5:**
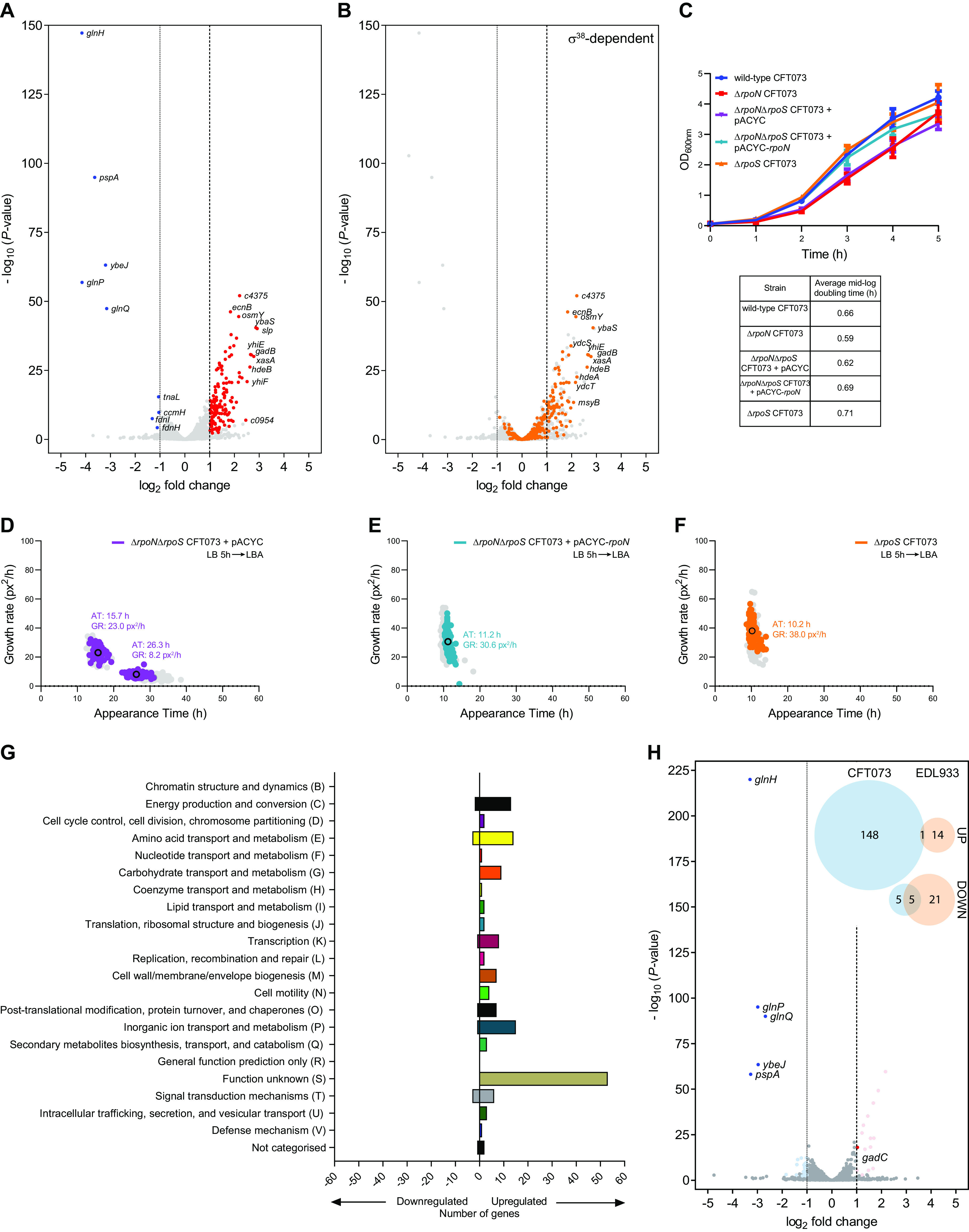
Comparative analysis of the transcriptomes of wild-type and *ΔrpoN* CFT073 strains. (A) Volcano plot showing differentially expressed genes in the Δ*rpoN* CFT073 strain as a log_2_ change from the wild-type CFT073 strain extracted from LB liquid medium during mid-exponential growth. Significantly differentially expressed genes (DEGs) were defined as having an absolute log_2_ change ≥ 1, and a false discovery rate adjusted *P* < 0.05. Upregulated DEGs are shown in red, downregulated DEGs are shown in blue, and the largest fold changes are labeled with gene names. (B) As in (A) but known σ^38^-dependent genes are highlighted in orange. Gene names of σ^38^-dependent genes with the highest fold change are labeled. (C) Growth curves of wild-type (blue), Δ*rpoN* (red), Δ*rpoN*Δ*rpoS* (purple), Δ*rpoN*Δ*rpoS* + *rpoN* (cyan), and Δ*rpoS* (orange) CFT073 strains grown in LB liquid medium for 5 h. Error bars represent standard deviation (*n* = 3). (D to F) Scanlag analysis as in Fig. 2 of (D) Δ*rpoN*Δ*rpoS* (purple), (E) Δ*rpoN*Δ*rpoS* + *rpoN* (cyan), and (F) Δ*rpoS* (orange) CFT073 strains grown in LB liquid medium for 5 h before plating on LB agar (LBA) plates. Black circles represent the average population growth rate and appearance time with the mean growth rate indicated as a value. Shown in gray for comparison are the M1 and M2 colonies of Δ*rpoN* CFT073 (in (D)) and wild-type (in (E) and (F)) bacteria grown in LB liquid medium for 5 h before plating. (G) DEGs in the Δ*rpoN* CFT073 bacteria from (A) were categorized by clustering of orthologous groups (COG) annotation. (H) As in (A) but for DEGs in Δ*rpoN* EDL933 bacteria as a log_2_ change from wild-type EDL933 bacteria extracted of Δ*rpoN* CFT073 (in (D)) and wild-type (in (E) and (F)) LB liquid medium during mid-exponential growth. Upregulated DEGs are shown in pale red and downregulated DEGs shown in pale blue, with DEGs also present in (A) labeled and shown in bright red and bright blue, respectively. The Venn diagrams (inset) show upregulated and downregulated DEG numbers from Δ*rpoN* CFT073 bacteria (blue) compared to Δ*rpoN* EDL933 bacteria (orange) with numbers in overlapping circles representing identical genes in both strains.

To better understand the requirement of σ^54^ for uniform colony growth of E. coli CFT073, we compared the transcriptomes of exponentially growing wild-type, Δ*rpoN* and Δ*rpoN* containing plasmid pACYC-*rpoN* bacteria. We defined differentially expressed genes as those with expression levels changed ≥2-fold with a false discovery rate adjusted *P* < 0.05. A total of 159 genes were differentially expressed in the Δ*rpoN* CFT073 strain (Table S2 and Fig. 5A), of which 149 (2.7% of total genes) were upregulated and 10 (0.2% of total genes) were downregulated. Of the genes that were downregulated, *glnHPQ*, *ybeJ*, and *pspA* are transcribed from known σ^54^-dependent promoters. The products of *glnHPQ*, *ybeJ*, and *pspA* are involved in nitrogen and membrane stress responses, and their transcription is dependent on the activator ATPases, NtrC and PspF, respectively. To the best of our knowledge, E. coli cells growing exponentially in LB liquid medium do not experience any nitrogen limitation or membrane stress. Therefore, it is unlikely that the downregulation of *glnHPQ*, *ybeJ,* and/or *pspA* underpins the heterogeneous appearance of Δ*rpoN* CFT073 colonies on LB agar plates. The unknown gene product, *c0897*, was categorized as significantly downregulated, but genomic visualization revealed that this was due to its overlap the *glnHPQ* promoter, and the gene product itself was not differentially expressed and was omitted from downstream analysis. We next focused on the genes that are upregulated in the Δ*rpoN* CFT073 strain. As shown in Fig. 5B, we noted that the majority (76 out of 149) of the conserved upregulated genes in the Δ*rpoN* CFT073 strain either belonged to or are associated with the *rpoS* regulon (as defined in the non-pathogenic E. coli strain MG1655 (19, 20)) and transcribed by the general stress response σ factor, σ^38^ (product of *rpoS*). The functional interconnection between the *rpoN* and *rpoS* regulons is undisputed as the absence of *rpoN* has been shown to lead to either increased σ^38^ levels or σ^38^ stability in both enterohemorrhagic and non-pathogenic E. coli strains (7, 8, 21). Therefore, we considered whether the heterogeneous appearance of Δ*rpoN* CFT073 colonies was linked to increased σ^38^ activity. To investigate this, we constructed Δ*rpoS* and Δ*rpoN*Δ*rpoS* CFT073 strains and compared their growth properties in LB liquid medium and by Scanlag analysis. As shown in Fig. 5C, the doubling time of the Δ*rpoN*Δ*rpoS* CFT073 strain did not differ from that of the Δ*rpoN* CFT073 strain. Further, the doubling time of the Δ*rpoS* CFT073 strain did not differ from that of the wild-type CFT073 strain. Notably, in the Scanlag experiment, the colonies of the Δ*rpoN*Δ*rpoS* CFT073 strain appeared in a heterogeneous fashion and their apparent growth rates and appearance times resembled that of those seen with the Δ*rpoN* CFT073 strain (Fig. 5D). However, we note that the M2 colonies of the Δ*rpoN*Δ*rpoS* CFT073 strain appeared faster (by ∼4 h) than the M2 colonies of the Δ*rpoN* CFT073 strain. As expected, the colonies of the Δ*rpoN*Δ*rpoS* CFT073 strain containing plasmid pACYC-*rpoN* resembled wild-type colonies (Fig. 5E). Further, the colonies of the Δ*rpoS* CFT073 strain did not grow in a heterogeneous fashion and resembled wild-type colonies (Fig. 5F). Overall, it seems that *rpoS* does not directly contribute to the heterogeneous appearance of Δ*rpoN* CFT073 colonies. Because many of the derepressed genes have no assigned function (Fig. 5G), at this stage in our analysis, it was difficult to delineate the genetic pathways that contribute to the heterogeneous appearance of Δ*rpoN* CFT073 colonies in greater granularity. However, as shown in Fig. 2H, the colonies of the Δ*rpoN* EDL933 strain did not appear in a heterogeneous manner. Therefore, we compared the transcriptomes of exponentially growing wild-type, Δ*rpoN* and Δ*rpoN* + pACYC-*rpoN* EDL933 bacteria and observed that a total of 41 genes were differentially expressed in the Δ*rpoN* EDL933 strain (Table S3 and Fig. 5H). Of these, 15 (0.3% of total genes) were upregulated and 26 (0.5% of total genes) were downregulated. Most of the upregulated genes were associated with amino acid metabolism (histidine and threonine) and many of the genes downregulated in the Δ*rpoN* CFT073 and EDL933 strains were the same (i.e., *glnH*, *glnP*, *glnQ*, *ybeJ*, and *pspA*). However, of the 15 upregulated genes in the Δ*rpoN* EDL933 strain, only 1 was commonly upregulated between the two Δ*rpoN* mutant strains (Fig. 5H inset) – *gadC*, which encodes an antiporter as part of the glutamate-dependent acid resistance system. Notably, while many of the σ^38^-dependent acid resistance system genes are upregulated in the Δ*rpoN* CFT073 strain, no other associated genes were similarly upregulated in the Δ*rpoN* EDL933 strain. Overall, it seems that under our experimental conditions, where σ^54^-dependent transcription is not known to be essential, and the absence of *rpoN* in CFT073 and EDL933 E. coli strains leads to the derepression of different sets of genes. In the case of the CFT073 strain, this derepression of gene expression leads to heterogeneous colony growth. It seems that, under conditions where σ^54^-dependent transcription is not essential, σ^54^ has a non-canonical regulatory function in transcriptionally repressing gene expression.

### Activator ATPases do not appear to contribute to σ^54^’s role as a determinant of uniform colony growth of E. coli CFT073 strain.

**FIG 6:**
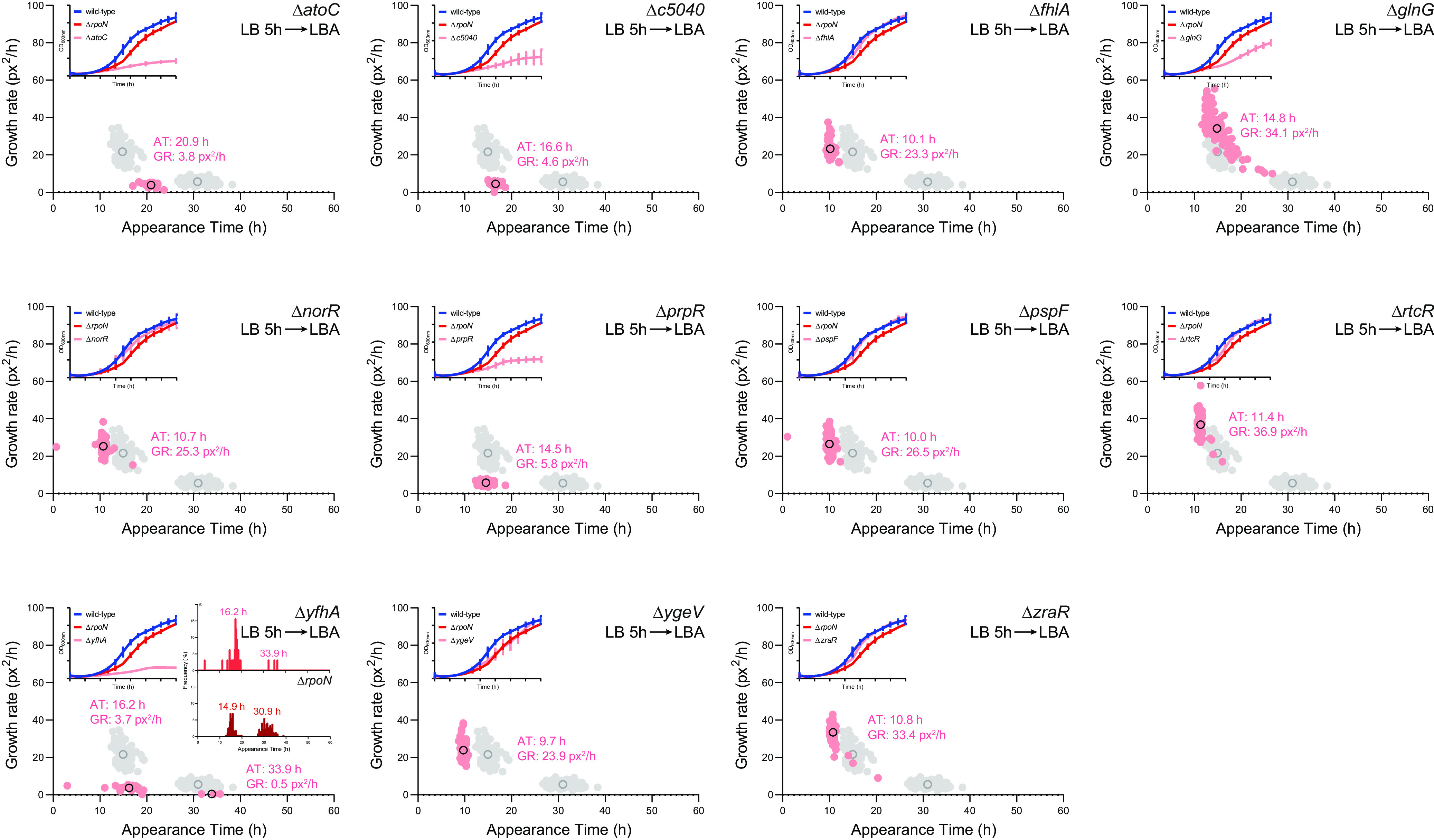
Activator ATPases do not appear to contribute to σ^54^’s role as a determinant of uniform colony growth of E. coli CFT073. Scanlag analysis as in Fig. 2 following growth in LB liquid medium for 5 h of known activator ATPase mutants of CFT073 bacteria in order from top left: Δ*atoC*, Δ*c5040*, Δ*fhlA*, Δ*glnG*, Δ*norR*, Δ*prpR*, Δ*pspF*, Δ*rtcR*, Δ*yfhA*, Δ*ygeV*, and Δ*zraR*. Black circles represent the average population growth rate and appearance time with the mean growth rate indicated as a value. Shown in gray for comparison are the M1 and M2 colonies of Δ*rpoN* CFT073 bacteria grown in LB liquid medium for 5 h before plating. The inset graphs show growth curves of each activator ATPase mutant strain (pink) grown in LB for 5h with growth curves of wild-type (blue) and Δ*rpoN* (red) CFT073 strains shown for comparison. Inset histograms for the Δ*yfhA* CFT073 strain show appearance times of Δ*yfhA* (top, pink) and Δ*rpoN* (bottom, red) for comparison (see the text) with average population appearance time written above each peak. Where indicated, the error bars represent standard deviation (*n* = 3).

To further establish the potential non-canonical regulatory function of σ^54^, we focused on the activator ATPases. Recall that the σ^54^-RNAP requires specialized activator ATPases for its canonical function as a promoter-specificity factor. A given activator ATPase is responsible for activating transcription of genes belonging to a defined regulon. Therefore, it was unlikely that more than one activator ATPase is involved in the transcription of genes that belong to a defined regulon. In other words, the activator ATPases often confer narrow regulon specificity. Hence, our rationale was that, if the heterogeneous appearance of the Δ*rpoN* CFT073 colonies was due to a canonical function of σ^54^, then a CFT073 strain lacking anyone of the activator ATPases would also phenocopy the Δ*rpoN* CFT073 strain in the ScanLag assay. Conversely, this would not be the case if a non-canonical regulatory function of σ^54^ was responsible for the heterogeneous appearance of the Δ*rpoN* CFT073 colonies. Hence, we searched the CFT073 genome for proteins that have the hallmark ‘GAFTGA’ sequence of activator ATPases required for interacting with the σ^54^-RNAP and identified 11 activators ATPases. As shown in Fig. 6 (insets), the 11 activator ATPase mutant strains of CFT073 can be classified into two classes based on their growth properties in LB liquid medium in a plate reader incubator at 37°C: the *ΔatoC*, *ΔyfhA*, *ΔprpR*, *ΔglnG*, and Δ*c5040* displayed markedly compromised growth compared to the wild-type, *ΔrpoN*, and other activator ATPase mutant strains. Thus, for the ScanLag experiment, we grew each activator ATPase mutant strain for 5 h, by which time, all had reached at least early exponential-phase of growth and, based on optical density correction, plated approximately equal numbers of bacteria onto LB agar plates. The results showed that, for each activator ATPase mutant CFT073 strain, the differences in growth observed in LB liquid medium were reflected in the apparent growth rate of the colonies on LB agar plates (Fig. 6). However, we did not detect the characteristic heterogeneous appearance of colonies, as seen with the Δ*rpoN* CFT073 strain, for any of the activator ATPase mutant CFT073 strains. We noted that some *ΔyfhA* colonies resembled the M2 colonies of the *ΔrpoN* strain but, unlike the M2 colonies of the *ΔrpoN* strain, the frequency of the slower growing colonies of the *ΔyfhA* strain represented < 10% of the total number of colonies on the plate. Our results, therefore, indicate that the absence of any one of the activator ATPases encoded by the CFT073 strain does not phenocopy the *ΔrpoN* CFT073 strain. Overall, (i) as activator ATPases respond to a single signal to activate their respective regulons, and (ii) under conditions used here, we are not aware of any signal that would trigger any signaling cascade to activate σ^54^-dependent regulons, the results in Fig. 6 support the view that the heterogeneous appearance of *ΔrpoN* CFT073 colonies is due to a potential non-canonical regulatory function of σ^54^ in the CFT073 strain.

## DISCUSSION

The σ subunits of the bacterial RNAP are central regulators of gene expression because they confer promoter-specificity upon the RNAP and thereby determine the expression of different regulons. The σ^54^ factor is distinct from other bacterial σ factors as it has an obligatory requirement for an activator ATPase to allow transcription by its RNAP. Therefore, different activator ATPases determine the cognate regulon specificity of the σ^54^-RNAP in response to diverse environmental cues. This study has revealed a role for σ^54^ in the UPEC strain CFT073 as a determinant for uniform colony growth and, consequently, the absence of σ^54^ results in a heterogeneous appearance of colonies consisting of two subpopulations, both of which appear slower than the wild-type population on LB solid media. Under conditions used here, the absence of σ^54^ does not seem to affect the pathogenic capacity of E. coli CFT073 bacteria, but our results indicated that the phenotype of the mutant bacteria imposes potentially adverse fitness consequences on the mutant population under growth-restrictive conditions on solid media. We speculate that the mutant bacteria will be equally compromised for growth on foreign indwelling surfaces (e.g., catheters) in the urinary tract, which are often impregnated or coated with antibacterial agents.

Although we are unable to specify the genetic basis by which σ^54^ determines uniform colony growth of the E. coli CFT073 strain, notably, the absence of σ^54^ results in the transcriptional upregulation of a subset of seemingly functionally unconnected genes. Intriguingly, previous studies by Schaefer et al. (22) and Shimada et al. (23) in non-pathogenic *E. coli* strain BW25113 and W3350, respectively, implied that the σ^54^ or its RNAP could have a repressive effect on global transcription. It is thus possible that the transcriptionally attenuated RPc formed by the σ^54^-RNAP (see Introduction) when not required to be activated by the activator ATPase, could have an indirect regulatory function in controlling genetic information flow. Further, a study by Bonocora et al. (24) identified 85 intragenic binding events by the σ^54^-RNAP mostly within σ^54^-independent genes in the genome of nonpathogenic E. coli strain MG1655 under a growth condition not associated with the activity of any activator ATPases, suggesting that most, if not all, of these promiscuous binding events, could contribute to the transcriptional program differently. Our results unequivocally indicate that σ^54^’s role as a determinant of uniform colony growth was not dependent on any of the individual activator ATPases present in the E. coli CFT073 strain. This is unsurprising because, to the best of our knowledge, the exponential growth of E. coli in LB liquid medium or LB solid media, does not require σ^54^. Hence, we propose that the requirement for σ^54^ for uniform colony growth represents a non-canonical function of σ^54^ in the regulation of gene expression in bacteria. As σ^54^ is the only alternative σ factor that is abundant in E. coli during the exponential-phase of growth in LB liquid medium (i.e., the condition used here) (25), we envisage that σ^54^ functions like a nucleoid-associated protein (NAP) and thereby represses the transcription of to allow uniform colony growth by the E. coli CFT073 strain. The observation that the absence of σ^54^ also results in the transcriptional upregulation of genes in the E. coli EDL933 strain, albeit not resulting in any heterogeneous effect on colony growth, further supporting a potential NAP-like function for σ^54^ in transcription regulation. Notably, different genes become transcriptionally upregulated in Δ*rpoN* CFT073 and Δ*rpoN* EDL933 bacteria, suggesting that the non-canonical regulatory capacity of σ^54^ varies between strains of the same species. This might also explain why the absence of σ^54^ in either the EDL933 or NCM3722 E. coli strains does not result in the heterogeneous colony appearance phenotype on LB agar plates. Finally, in support of the potential NAP-like function for σ^54^, the Δ*rpoN* CFT073 strain harboring the plasmid pACYC-*rpoN* encoding a σ^54^ variant containing the R456A substitution, which abrogates σ^54^’s ability to bind DNA but not RNAP (26), phenocopies the Δ*rpoN* CFT073 strain in the ScanLag assay (Fig. S1). Although, we point out a potential caveat of the interpretation of these data is that the σ^54^ variant with the R456A substitution is also defective for its canonical function (which is unlikely to be involved under our conditions). It is difficult to decipher whether the potential NAP-like function of σ^54^ is mediated by σ^54^ per se or in the context of the RNAP. The former is a possibility as σ^54^, unlike any other six σ subunits in E. coli, can bind DNA independently of the RNAP (27).

Bacterial σ factors are considered general transcription factors. In a recent review, Dorman et al. (28) proposed assigning a transcription regulatory protein as a ‘transcription factor’ or ‘NAP’ should be considered ad hoc operational definitions. Hence, the potential NAP-like function of σ^54^ implied from our work, which clearly warrants detailed mechanistic investigation, further underscores this point and highlights the mechanistic complexity underpinning transcription regulation in bacteria.

## MATERIALS AND METHODS

### Bacterial strains and plasmids.

Escherichia coli strains and plasmids used in this study are listed in Table S1. The Δ*rpoN* mutants for all strains were made according to the λ Red recombinase method to replace the *rpoN* gene with an in-frame fusion encoding a kanamycin resistance cassette amplified from the pDOC-K plasmid (29). The Δ*rpoN*Δ*rpoS* CFT073 mutant strain was generated using the same recombinase method by replacing the *rpoS* gene with an in-frame fusion encoding a kanamycin-resistant cassette using the Δ*rpoN* CFT073 strain as the parent strain. All mutant strains were cured of their kanamycin resistance cassette using the pCP20 plasmid (30). The *rpoN* complementation plasmid was constructed using Gibson assembly (31) to introduce the *rpoN* gene and ∼300 bp of the upstream native sequence (including the promoter) into a modified pACYC184 plasmid backbone (see Table S1 for details).

### Bacterial growth.

The planktonic growth assays were conducted in lysogeny broth (LB) liquid media, filtered healthy female urine (taken from midstream flow), or LB containing 25% (vol/vol) human serum in 25 mL flasks (or 96-well plates for the activator ATPase mutant growth curves) at 37°C, shaking at ∼180 rpm. Growth curves were produced by measuring the optical density at 600 nm (OD_600_) of bacterial cultures as a function of time. All growth curves shown in figures represent the mean average from at least three biological replicates with the standard error of the mean (SEM) plotted as error bars. The doubling time in the mid-log phase for growth curves was calculated according to Powell (32). Viability measurements were conducted by plating serial dilutions of cultures at relevant time points and counting CFU per mL of culture.

### Scanlag assays.

Aliquots of bacterial cultures grown as described above were taken at time points indicated in the text, washed twice in sterile phosphate-buffered saline, diluted between 10^−5^ to 10^−6^, and 100 μL spread on either LB agar plates with or without specified antibiotics (2 μg/mL amikacin disulfate salt or 7 μg/mL nitrofurantoin [both Sigma-Aldrich]) or 50% (vol/vol) healthy female human urine containing 1.5% (wt/vol) agar. Plates were incubated at 33°C in a standard office scanner (Epson Perfection V370 photo scanner, J232D) placed in an incubator, and images were taken every 20 min over a 48-h period. Analysis of appearance time and apparent growth rate of colonies was adapted from Levin-Reisman et al. (18) using a modified code by Miles Priestman available at https://github.com/mountainpenguin/NQBMatlab. At least two independent experiments were conducted for each ScanLag experiment shown in the figures.

### Cell culture infection assays.

The HBT-9 5637 human bladder cell line (ATCC) was used for infection assays. Cells were cultured in RPMI 1640 medium (ATCC) supplemented with 10% (vol/vol) fetal calf serum (FCS) (Sigma) and seeded overnight in 24-well cell culture plates. Bacterial strains were grown overnight in RPMI 1640 medium supplemented with 10% FCS, before washing with phosphate-buffered saline (PBS) and resuspending in fresh RPMI 1640 + 10% FCS. Cells (HBT-9 5637) were washed and aspirated three times in PBS before bacterial strains were added at a multiplicity of infection (MOI) of 50. Plates were incubated for 2 h at 37°C, 5% CO_2_. Adhered + internalized bacteria were extracted by washing and aspirating medium five times with PBS and adhered + internalized bacteria were released from wells following treatment with 0.1% (vol/vol) Triton X-100 (Merck) for 5 min at 37°C to kill the HBT-9 5637 cells and release the bacteria. Total bacteria were extracted in the same way, but the medium was also kept to account for any planktonic (i.e., nonadhered) bacteria. Internalized only bacteria were also extracted in the same way, but cells were treated for 1 h with 100 μg/mL gentamicin sulfate (Sigma) to remove any planktonic or adherent bacteria. Bacteria were plated for either CFU counts or ScanLag as detailed above.

### RNA sequencing.

Cultures were grown in LB liquid medium and sampled during mid-exponential phase (OD_600_ = 1). Three biological replicates of each strain were taken and mixed with a phenol:ethanol (1:19) solution at a ratio of 9:1 (culture:solution) before harvesting the bacteria immediately by centrifugation. Bacterial pellets were sent to Vertis Biotechnologie AG for downstream processing. Briefly, RNA was extracted from the bacterial pellets using the RNAsnap (33) protocol followed by depletion of rRNA (rRNA) species with the bacterial Ribo-Zero rRNA removal kit (Epicentre). RNase III was used to fragment the remaining RNA, followed by poly(A)-tailing of samples and tobacco acid pyrophosphatase treatment (TAP; Epicentre). cDNA synthesis was conducted with oligonucleotide (dT)-adapter primers and M-MLV reverse transcriptase, followed by PCR amplification using TruSeq-designed primers from Illumina guidelines. cDNA was sequenced with an Illumina NextSeq 500. Downstream data analysis was performed using standard parameters on the CLC Genomics Workbench 11. RNA-seq reads for CFT073 and EDL933 genomes were mapped using Burrows-Wheeler Aligner to E. coli CFT073 (AE014075) and EDL933 (NZ_CP008957.1) strains, respectively. Unique reads mapped and total read counts were used for data normalization. Reads mapped to each gene were quantified to give a matrix of read counts, which was then analyzed with the DESeq2 BioConductor package to identify differentially expressed genes. Genes with ≤10 reads mapped were excluded. All statistical analysis for differential gene expression was conducted with R version 4.1.1. Sequencing data are available in the ArrayExpress database at EMBL-EBI (http://www.ebi.ac.uk/arrayexpress) under accession number E-MTAB-11288.

### Data availability.

Data will be shared upon request to the corresponding author, Sivaramesh Wigneshweraraj.

## References

[1] Paget MS. 2015. Bacterial sigma factors and anti-sigma factors: structure, function and distribution. Biomolecules 5:1245–1265. 10.3390/biom5031245. 26131973 PMC4598750

[2] Hirschman J, Wong PK, Sei K, Keener J, Kustu S. 1985. Products of nitrogen regulatory genes ntrA and ntrC of enteric bacteria activate glnA transcription in vitro: evidence that the ntrA product is a sigma factor. Proc Natl Acad Sci USA 82:7525–7529. 10.1073/pnas.82.22.7525. 2999766 PMC390849

[3] Wigneshweraraj S, Bose D, Burrows PC, Joly N, Schumacher J, Rappas M, Pape T, Zhang X, Stockley P, Severinov K, Buck M. 2008. Modus operandi of the bacterial RNA polymerase containing the sigma54 promoter-specificity factor. Mol Microbiol 68:538–546. 10.1111/j.1365-2958.2008.06181.x. 18331472

[4] Wigneshweraraj SR, Burrows PC, Bordes P, Schumacher J, Rappas M, Finn RD, Cannon WV, Zhang X, Buck M. 2005. The second paradigm for activation of transcription. Prog Nucleic Acids Res Mol Biol 79:339–369. 10.1016/S0079-6603(04)79007-8. 16096032

[5] Reitzer L, Schneider BL. 2001. Metabolic context and possible physiological themes of sigma(54)-dependent genes in Escherichia coli. Microbiol Mol Biol Rev 65:422–444. table of contents. 10.1128/MMBR.65.3.422-444.2001. 11528004 PMC99035

[6] Riordan JT, Mitra A. 2017. Regulation of Escherichia coli pathogenesis by alternative sigma factor N. EcoSal Plus 7. 10.1128/ecosalplus.ESP-0016-2016. PMC11575691 28635589

[7] Mitra A, Fay PA, Vendura KW, Alla Z, Carroll RK, Shaw LN, Riordan JT. 2014. Sigma(N) -dependent control of acid resistance and the locus of enterocyte effacement in enterohemorrhagic Escherichia coli is activated by acetyl phosphate in a manner requiring flagellar regulator FlhDC and the sigma(S) antagonist FliZ. Microbiologyopen 3:497–512. 10.1002/mbo3.183. 24931910 PMC4287178

[8] Mitra A, Fay PA, Morgan JK, Vendura KW, Versaggi SL, Riordan JT. 2012. Sigma factor N, liaison to an ntrC and rpoS dependent regulatory pathway controlling acid resistance and the LEE in enterohemorrhagic Escherichia coli. PLoS One 7:e46288. 10.1371/journal.pone.0046288. 23029465 PMC3459932

[9] Riordan JT, Tietjen JA, Walsh CW, Gustafson JE, Whittam TS. 2010. Inactivation of alternative sigma factor 54 (RpoN) leads to increased acid resistance, and alters locus of enterocyte effacement (LEE) expression in Escherichia coli O157: H7. Microbiology (Reading) 156:719–730. 10.1099/mic.0.032631-0. 19942657 PMC2889430

[10] Asadi Karam MR, Habibi M, Bouzari S. 2019. Urinary tract infection: pathogenicity, antibiotic resistance and development of effective vaccines against Uropathogenic Escherichia coli. Mol Immunol 108:56–67. 10.1016/j.molimm.2019.02.007. 30784763

[11] Kot B. 2019. Antibiotic resistance among uropathogenic Escherichia coli. Pol J Microbiol 68:403–415. 10.33073/pjm-2019-048. 31880885 PMC7260639

[12] Frick-Cheng AE, Sintsova A, Smith SN, Krauthammer M, Eaton KA, Mobley HLT. 2020. The gene expression profile of uropathogenic Escherichia coli in women with uncomplicated urinary tract infections is recapitulated in the mouse model. mBio 11:e01412-20. 10.1128/mBio.01412-20. 32788379 PMC7439467

[13] Sintsova A, Frick-Cheng AE, Smith S, Pirani A, Subashchandrabose S, Snitkin ES, Mobley H. 2019. Genetically diverse uropathogenic Escherichia coli adopt a common transcriptional program in patients with UTIs. Elife 8:e49748. 10.7554/eLife.49748. 31633483 PMC6802966

[14] Snyder JA, Haugen BJ, Buckles EL, Lockatell CV, Johnson DE, Donnenberg MS, Welch RA, Mobley HL. 2004. Transcriptome of uropathogenic Escherichia coli during urinary tract infection. Infect Immun 72:6373–6381. 10.1128/IAI.72.11.6373-6381.2004. 15501767 PMC523057

[15] Hagan EC, Lloyd AL, Rasko DA, Faerber GJ, Mobley HL. 2010. Escherichia coli global gene expression in urine from women with urinary tract infection. PLoS Pathog 6:e1001187. 10.1371/journal.ppat.1001187. 21085611 PMC2978726

[16] Kao JS, Stucker DM, Warren JW, Mobley HL. 1997. Pathogenicity island sequences of pyelonephritogenic Escherichia coli CFT073 are associated with virulent uropathogenic strains. Infect Immun 65:2812–2820. 10.1128/iai.65.7.2812-2820.1997. 9199454 PMC175396

[17] Soupene E, van Heeswijk WC, Plumbridge J, Stewart V, Bertenthal D, Lee H, Prasad G, Paliy O, Charernnoppakul P, Kustu S. 2003. Physiological studies of Escherichia coli strain MG1655: growth defects and apparent cross-regulation of gene expression. J Bacteriol 185:5611–5626. 10.1128/JB.185.18.5611-5626.2003. 12949114 PMC193769

[18] Levin-Reisman I, Fridman O, Balaban NQ. 2014. ScanLag: high-throughput quantification of colony growth and lag time. J Vis Exp 89:51456. 10.3791/51456. PMC4215631 25077667

[19] Weber H, Polen T, Heuveling J, Wendisch VF, Hengge R. 2005. Genome-wide analysis of the general stress response network in Escherichia coli: sigmaS-dependent genes, promoters, and sigma factor selectivity. J Bacteriol 187:1591–1603. 10.1128/JB.187.5.1591-1603.2005. 15716429 PMC1063999

[20] Wong GT, Bonocora RP, Schep AN, Beeler SM, Lee Fong AJ, Shull LM, Batachari LE, Dillon M, Evans C, Becker CJ, Bush EC, Hardin J, Wade JT, Stoebel DM. 2017. Genome-wide transcriptional response to varying RpoS levels in Escherichia coli K-12. J Bacteriol 199:e00755-16. 10.1128/JB.00755-16. 28115545 PMC5350281

[21] Dong T, Yu R, Schellhorn H. 2011. Antagonistic regulation of motility and transcriptome expression by RpoN and RpoS in Escherichia coli. Mol Microbiol 79:375–386. 10.1111/j.1365-2958.2010.07449.x. 21219458

[22] Schaefer J, Engl C, Zhang N, Lawton E, Buck M. 2015. Genome wide interactions of wild-type and activator bypass forms of sigma54. Nucleic Acids Res 43:7280–7291. 10.1093/nar/gkv597. 26082500 PMC4551910

[23] Shimada T, Furuhata S, Ishihama A. 2021. Whole set of constitutive promoters for RpoN sigma factor and the regulatory role of its enhancer protein NtrC in Escherichia coli K-12. Microb Genom 7:000653. 10.1099/mgen.0.000653. PMC8743547 34787538

[24] Bonocora RP, Smith C, Lapierre P, Wade JT. 2015. Genome-scale mapping of Escherichia coli sigma54 reveals widespread, conserved intragenic binding. PLoS Genet 11:e1005552. 10.1371/journal.pgen.1005552. 26425847 PMC4591121

[25] Ishihama A. 2000. Functional modulation of Escherichia coli RNA polymerase. Annu Rev Microbiol 54:499–518. 10.1146/annurev.micro.54.1.499. 11018136

[26] Wang L, Gralla JD. 2001. Roles for the C-terminal region of sigma 54 in transcriptional silencing and DNA binding. J Biol Chem 276:8979–8986. 10.1074/jbc.M009587200. 11124262

[27] Buck M, Cannon W. 1992. Specific binding of the transcription factor sigma-54 to promoter DNA. Nature 358:422–424. 10.1038/358422a0. 1641025

[28] Dorman CJ, Schumacher MA, Bush MJ, Brennan RG, Buttner MJ. 2020. When is a transcription factor a NAP? Curr Opin Microbiol 55:26–33. 10.1016/j.mib.2020.01.019. 32120333 PMC8048100

[29] Lee DJ, Bingle LE, Heurlier K, Pallen MJ, Penn CW, Busby SJ, Hobman JL. 2009. Gene doctoring: a method for recombineering in laboratory and pathogenic Escherichia coli strains. BMC Microbiol 9:252. 10.1186/1471-2180-9-252. 20003185 PMC2796669

[30] Cherepanov PP, Wackernagel W. 1995. Gene disruption in Escherichia coli: TcR and KmR cassettes with the option of Flp-catalyzed excision of the antibiotic-resistance determinant. Gene 158:9–14. 10.1016/0378-1119(95)00193-A. 7789817

[31] Gibson DG, Young L, Chuang RY, Venter JC, Hutchison CA, 3rd, Smith HO. 2009. Enzymatic assembly of DNA molecules up to several hundred kilobases. Nat Methods 6:343–345. 10.1038/nmeth.1318. 19363495

[32] Powell EO. 1956. Growth rate and generation time of bacteria, with special reference to continuous culture. J Gen Microbiol 15:492–511. 10.1099/00221287-15-3-492. 13385433

[33] Stead MB, Agrawal A, Bowden KE, Nasir R, Mohanty BK, Meagher RB, Kushner SR. 2012. RNAsnap: a rapid, quantitative and inexpensive, method for isolating total RNA from bacteria. Nucleic Acids Res 40:e156. 10.1093/nar/gks680. 22821568 PMC3488207

