# Supplementary material for: A Role for the RNA Polymerase Gene Specificity Factor σ54 in the Uniform Colony Growth of Uropathogenic Escherichia coli
Source: J Bacteriol. 2022 Mar 31;204(4):e00031-22. doi: 10.1128/jb.00031-22 (PMC9017345; doi:10.1128/jb.00031-22)
Supplement: Supplemental file 1 — Fig. S1, Tables S1 to S3. Download jb.00031-22-s0001.pdf, PDF file, 0.3 MB [file jb.00031-22-s0001.pdf]

Supplementary material

A role for the RNA polymerase gene specificity factor σ^{54} in the uniform colony growth of uropathogenic *Escherichia coli*

Amy Switzer*, Lynn Burchell, Panagiotis Mitsidis, Teresa Thurston, and Sivaramesh Wigneshweraraj*

MRC Centre for Molecular Bacteriology and Infection, Imperial College London, London, SW7 2AZ, UK

Figure S1

Table S1

Table S2

Table S3

Figure S1

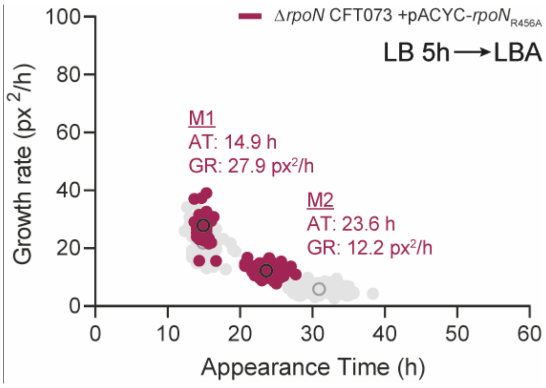

Figure S1. Scanlag analysis of colony appearance time (AT; h) and growth rate in pixels^2/h (GR; px^2/h) for Δ*rpoN* + pACYC-*rpoN*$_{R456A}$ CFT073 bacteria grown in LB liquid media for 5 h and plated onto LB agar (LBA) plates. Black circles represent average population growth rate and appearance time with mean average values indicated. Liquid to solid growth conditions are indicated in the top right. Shown in grey for comparison are the M1 and M2 colonies from Δ*rpoN* CFT073 bacteria grown in LB liquid media for 5 h before plating.

Table S1

E. coli strains and plasmids used in this study

Strains		
Name	Description	Source or Reference
Wild-type CFT073	UPEC O6:K2:H1 WAM4505, original patient isolate	Gifted from Rodney Welch (1)
Δ*rpoN* CFT073	UPEC O6:K2:H1 WAM4505, *ΔrpoN*	This study
Δ*rpoN* CFT073 + pACYC-*rpoN*	UPEC O6:K2:H1 WAM4505, *ΔrpoN* + pACYC-*rpoN*	This study
Wild-type EDL933	EHEC O157:H7 strain, EDL933	(2)
ΔrpoN EDL933	EHEC O157:H7 strain, EDL933 *ΔrpoN*	This study
Δ*rpoN* EDL933 + pACYC-*rpoN*	EHEC O157:H7 strain, EDL933 *ΔrpoN* + pACYC-*rpoN*	This study
Wild-type NCM3722	Prototrophic K-12 strain, NCM3722	(3)
ΔrpoN NCM3722	Prototrophic K-12 strain, NCM3722 *ΔrpoN*	This study
Δ*rpoN* NCM3722 + pACYC-*rpoN*	Prototrophic K-12 strain, NCM3722 *ΔrpoN* + pACYC-*rpoN*	This study

Plasmids		
Name	Description	Source or reference
pACYC-*rpoN*	Modified pACYC184 plasmid backbone (-TcR, +MCS) expressing *rpoN* under the native *rpoN* promoter	This study

Table S2

Differentially expressed genes in Δ*rpoN* CFT073 bacteria relative to wild-type CFT073 bacteria

Gene	c #	Product name	**Log_2 fold change** (Δ*rpoN* / wild-type)	**Adjusted P-value**	**Log_2 fold change** (Δ*rpoN* + pACYC-*rpoN* / wild-type)	**Adjusted P-value**
slp	c4304	Outer membrane protein slp precursor	2.92	3.22E-38	0.34	0.4841
ybaS	c0605	Probable glutaminase ybaS	2.86	1.63E-38	0.16	NA
xasA	c1921	Amino acid antiporter	2.78	2.08E-28	0.01	NA
gadB	c1922	Glutamate decarboxylase beta	2.68	6.31E-29	0.02	0.9894
yhiE	c4323	Hypothetical protein yhiE	2.65	4.80E-29	0.52	NA
hdeB	c4320	Protein hdeB precursor	2.63	1.10E-24	0.26	0.5978
yhiF	c4306	Hypothetical transcriptional regulator yhiF	2.51	1.45E-19	0.30	NA
c0954	c0954	Putative capsid completion protein	2.46	3.25E-06	0.99	NA
yeaH	c2189	Hypothetical protein yeaH	2.30	6.82E-21	0.64	0.2019
hdeA	c4321	Protein hdeA precursor	2.21	3.01E-21	0.18	0.6931
c4375	c4375	Hypothetical protein yiaG	2.21	6.55E-50	0.46	0.2806
ybgS	c0829	Hypothetical protein ybgS precursor	2.19	1.02E-22	0.41	NA
osmY	c5457	Osmotically inducible protein Y precursor	2.18	1.70E-42	0.78	0.0858
ydcT	c1865	Hypothetical ABC transporter ATP-binding protein ydcT	2.16	2.53E-19	0.46	NA
c5459	c5459	Conserved hypothetical protein	2.08	7.76E-35	0.57	0.1831
msyB	c1318	Acidic protein msyB	2.08	2.30E-12	0.45	NA
ydcS	c1864	Putative ABC transporter Periplasmic binding protein ydcS precursor	1.98	4.20E-32	0.25	0.6699
ygaM	c3223	Hypothetical protein ygaM	1.96	1.94E-10	0.72	NA
hyaA	c1113	Hydrogenase-1 small chain precursor	1.94	8.32E-06	0.82	NA
c0950	c0950	Putative capsid scaffolding	1.91	6.05E-14	0.31	NA

		protein				
hdeD	c4322	HdeD protein	1.89	3.08E-19	0.25	0.5885
yhiU	c4324	Hypothetical lipoprotein yhiU precursor	1.88	1.57E-12	0.24	NA
yeaG	c2188	Hypothetical protein yeaG	1.88	6.31E-29	0.61	0.1079
narW	c1898	Respiratory nitrate reductase 2 delta chain	1.87	3.51E-06	0.22	NA
yahO	c0449	Hypothetical protein yahO precursor	1.86	4.50E-36	0.25	0.5545
ecnB	c5235	Putative toxin of osmotically regulated toxin-antitoxin system associated with programmed cell death	1.84	3.39E-44	0.44	0.3356
yehX	c2659	Hypothetical ABC transporter ATP-binding protein yehX	1.84	6.81E-13	0.61	NA
yegP	c2606	Hypothetical protein yegP	1.82	1.77E-31	0.53	0.2414
c2623	c2623	Fructose-bisphosphate aldolase class I	1.80	2.15E-28	0.48	0.1371
osmC	c1916	Osmotically inducible protein C	1.79	2.31E-19	0.46	0.3657
ydcV	c1867	Hypothetical ABC transporter permease protein ydcV	1.79	0.00038	-0.13	NA
ydiC	c2079	SufA protein	1.79	9.09E-09	0.64	NA
c0965	c0965	Phage baseplate assembly protein	1.76	3.50E-06	0.88	NA
yebV	c2245	Hypothetical protein yebV	1.75	3.09E-08	-0.06	NA
c1317	c1317	Hypothetical protein c1317	1.72	4.87E-08	0.45	NA
narU	c1901	Nitrite extrusion protein 2	1.69	6.49E-16	0.18	NA
yeaQ	c2200	Hypothetical protein yeaQ	1.68	4.67E-19	0.56	0.1920
ibpA	c4607	16 kDa heat shock protein A	1.68	1.26E-16	0.35	0.5843
narZ	c1900	Respiratory nitrate reductase 2 alpha chain	1.67	9.58E-21	0.20	0.7467
ynhD	c2077	Probable ATP-dependent transporter sufC	1.66	1.63E-09	0.67	NA
ynhC	c2076	SufD protein	1.64	5.25E-10	0.62	0.1202
tktB	c2990	Transketolase 2	1.64	5.15E-30	0.45	0.2301
poxB	c1004	Pyruvate dehydrogenase (cytochrome)	1.63	1.97E-27	0.52	0.2453
ydcU	c1866	Hypothetical ABC transporter permease protein ydcU	1.63	1.05E-08	0.34	NA
yehW	c2658	Hypothetical ABC transporter permease protein yehW	1.61	1.89E-11	0.21	NA
ynhE	c2078	SufB protein	1.60	1.43E-14	0.56	0.2463
c0952	c0952	Major capsid protein	1.60	2.23E-08	0.21	NA
ymgE	c1645	Transglycosylase associated	1.60	0.000148	0.80	NA

		protein				
otsB	c2311	Trehalose-phosphatase	1.59	6.43E-14	0.18	0.8361
c1843	c1843	Glyceraldehyde 3-phosphate dehydrogenase A	1.59	9.85E-16	0.23	0.7420
adhP	c1911	Alcohol dehydrogenase, propanol-preferring	1.57	3.51E-17	0.46	0.3991
ybaT	c0606	Hypothetical transport protein ybaT	1.56	4.67E-19	-0.16	0.7510
yhjY	c4366	Hypothetical protein yhjY	1.55	3.02E-15	0.20	0.8322
phnB	c5112	PhnB protein	1.55	1.16E-05	0.48	NA
narY	c1899	Respiratory nitrate reductase 2 beta chain	1.55	1.18E-09	-0.04	NA
wrbA	c1140	Flavoprotein wrbA	1.50	1.06E-10	0.71	0.0237
rpsV	c1913	30S ribosomal protein S22	1.50	1.26E-23	0.33	0.3327
ycaC	c1034	Protein ycaC	1.49	5.44E-25	0.21	0.7120
elaB	c2810	ElaB protein	1.49	3.07E-22	0.30	0.3589
fic	c4136	Cell filamentation protein fic	1.48	1.78E-08	0.51	NA
gabD	c3209	Succinate-semialdehyde dehydrogenase (NADP+)	1.48	9.45E-11	0.36	NA
blc	c5237	Outer membrane lipoprotein blc precursor	1.46	1.44E-24	0.49	0.3327
psiF	c0491	Phosphate starvation-inducible protein psiF precursor	1.45	8.30E-13	0.44	0.2972
c0936	c0936	Hypothetical protein c0936	1.44	3.14E-06	0.46	NA
c4407	c4407	Hypothetical protein c4407	1.44	5.47E-06	0.27	NA
yccJ	c1139	Hypothetical protein yccJ	1.42	9.30E-11	0.55	0.0765
bfr	c4107	Bacterioferritin	1.41	2.07E-25	0.33	0.4663
yehY	c2660	Hypothetical ABC transporter permease protein yehY	1.41	1.09E-11	0.08	NA
c2075	c2075	Selenocysteine lyase	1.40	1.61E-08	0.37	0.4498
ybiM	c0891	Hypothetical protein ybiM	1.39	0.000857	0.55	NA
yjbJ	c5016	Protein yjbJ	1.39	5.10E-26	0.29	0.5432
c0941	c0941	DNA adenine methylase	1.38	0.001938	0.75	NA
c0963	c0963	Putative Phage baseplate assembly protein	1.37	0.004379	0.36	NA
yccT	c1101	Hypothetical protein yccT precursor	1.36	4.47E-07	0.29	NA
yhiX	c4327	Transcriptional regulator gadX	1.36	8.12E-18	0.23	0.6790
yhfL	c4145	Hypothetical protein yhfL	1.35	0.031038	0.64	NA
ycgB	c1637	Hypothetical protein ycgB	1.34	3.44E-24	0.44	0.4016
yciR	c1756	Hypothetical protein yciR	1.34	2.95E-24	0.35	0.4294
ybaY	c0572	Hypothetical protein ybaY precursor	1.34	9.20E-19	0.34	0.3991
yqjE	c3857	Hypothetical protein yqjE	1.33	2.01E-18	0.42	0.1356

yhfG	c4137	Hypothetical protein yhfG	1.32	1.55E-10	0.66	0.2045
gabT	c3210	4-aminobutyrate aminotransferase	1.31	4.72E-11	0.25	NA
ynhA	c2074	SufE protein	1.30	0.007329	0.44	NA
yhcO	c3994	Hypothetical protein yhcO	1.30	1.24E-08	0.47	NA
ybhP	c0873	Hypothetical protein ybhP	1.30	7.77E-09	0.37	0.6150
katE	c2131	Catalase HPII	1.29	3.54E-10	0.29	0.6392
ydcJ	c1848	Hypothetical protein ydcJ	1.29	2.37E-09	0.09	NA
yqjK	c3858	Hypothetical protein yqjK	1.28	1.06E-23	0.30	0.2830
yjgB	c5370	Hypothetical zinc-type alcohol dehydrogenase-like protein yjgB	1.28	4.21E-13	0.32	0.4176
yehE	c2640	Hypothetical protein yehE precursor	1.28	5.23E-13	0.25	0.6931
yodD	c2372	Hypothetical protein yodD	1.27	4.46E-11	0.42	0.4153
otsA	c2310	Alpha,alpha-trehalose-phosphate synthase (UDP-forming)	1.27	4.58E-14	0.27	0.5653
glpA	c2782	Anaerobic glycerol-3-phosphate dehydrogenase subunit A	1.27	1.68E-08	0.11	0.9861
ggt	c4236	Gamma-glutamyltranspeptidase precursor	1.27	4.10E-13	0.17	0.7758
hyaB	c1114	Hydrogenase-1 large chain	1.27	0.001385	-0.09	NA
proP	c5116	Proline/betaine transporter	1.24	8.22E-16	0.67	0.1086
yqaE	c3215	Hypothetical protein yqaE	1.24	3.35E-11	0.28	0.6699
yehZ	c2661	Hypothetical protein yehZ precursor	1.24	1.34E-16	0.08	0.9273
gadA	c4328	Glutamate decarboxylase alpha	1.24	1.29E-08	-0.22	0.6392
glpB	c2783	Anaerobic glycerol-3-phosphate dehydrogenase subunit B	1.23	5.68E-08	0.63	0.8818
osmE	c2138	Osmotically inducible lipoprotein E precursor	1.23	4.59E-13	0.35	0.2972
glpD	c4203	Aerobic glycerol-3-phosphate dehydrogenase	1.23	5.73E-09	-0.01	0.9980
yjdI	c5133	Hypothetical protein yjdI	1.23	8.08E-05	0.45	NA
yebF	c2259	Hypothetical lipoprotein yebF precursor	1.22	8.96E-18	0.32	0.3474
glpC	c2784	Anaerobic glycerol-3-phosphate dehydrogenase subunit C	1.22	3.87E-05	0.79	0.8114
talA	c2989	Transaldolase A	1.22	8.54E-13	0.15	0.8114
ygaF	c3208	Hypothetical protein ygaF	1.21	1.87E-08	0.13	NA
ybdK	c0667	Hypothetical protein ybdK	1.20	3.24E-11	0.51	0.2295
yahK	c0447	Hypothetical zinc-type alcohol dehydrogenase-like	1.19	7.00E-10	0.23	0.6802

		protein yahK				
ydaM	c1815	Hypothetical protein ydaM	1.19	2.47E-12	0.14	0.8462
yliH	c0921	Hypothetical protein yliH	1.18	7.34E-08	0.21	NA
amyA	c2342	Cytoplasmic alpha-amylase	1.18	2.35E-13	0.31	0.5798
tam	c1942	Trans-aconitate 2-methyltransferase	1.18	1.10E-10	0.43	0.4294
coxT	c0935	Putative regulator for prophage	1.17	0.003387	0.48	NA
ygjG	c3828	Probable ornithine aminotransferase	1.17	2.81E-10	0.24	0.6931
ygaU	c3213	Unknown protein from 2D-page	1.15	1.36E-14	0.20	0.6932
yqjD	c3856	Hypothetical protein yqjD	1.15	2.28E-07	0.45	0.1488
yphA	c3065	Hypothetical protein yphA	1.15	8.83E-13	0.07	0.9473
chaB	c1677	Cation transport regulator chaB	1.14	7.88E-06	0.19	NA
yjdJ	c5134	Hypothetical protein yjdJ	1.13	1.92E-05	0.44	0.4115
ynhG	c2073	Hypothetical protein ynhG precursor	1.13	2.19E-07	0.54	0.1599
yegS	c2614	Hypothetical protein yegS	1.12	1.01E-08	0.02	0.9883
cysD	c3319	Sulfate adenylyltransferase subunit 2	1.11	0.045981	5.22	0.0136
yehV	c2657	MerR-like regulator A	1.11	3.98E-07	0.08	0.9597
cysJ	c3323	Sulfite reductase [NADPH] flavoprotein alpha-component	1.10	0.006736	4.62	0.0262
c3736	c3736	Putative enzyme	1.10	6.28E-05	0.03	NA
c0971	c0971	Probable major tail sheath protein	1.10	2.63E-05	0.08	NA
oppD	c1710	Oligopeptide transport ATP-binding protein oppD	1.09	2.28E-07	0.54	0.1183
c0948	c0948	Terminase, ATPase subunit	1.09	0.005215	-0.05	NA
ydhS	c2060	Hypothetical protein ydhS	1.09	5.82E-09	0.23	0.7582
yqjC	c3855	Protein yqjC precursor	1.09	1.93E-12	0.35	0.2355
sfaD	c1240	Putative minor F1C fimbrial subunit precursor	1.09	2.17E-06	0.20	0.7005
c4585	c4585	Conserved hypothetical protein	1.08	6.98E-05	-0.19	NA
ygaT	c3207	Hypothetical protein ygaT	1.08	0.012004	0.54	NA
aldB	c4408	Aldehyde dehydrogenase B	1.08	7.15E-06	-0.28	0.4948
ugpA	c4241	SN-glycerol-3-phosphate transport system permease protein ugpA	1.08	0.000505	-0.07	NA
aidB	c5275	AidB protein	1.08	1.44E-10	0.34	0.4912
yhiW	c4326	Hypothetical transcriptional regulator yhiW	1.08	2.58E-11	-0.20	0.7467
ydiZ	c2123	Hypothetical protein ydiZ	1.08	0.000261	0.28	NA
oppF	c1711	Oligopeptide transport ATP-binding protein oppF	1.07	7.27E-09	0.45	0.3447

focC	c1241	F1C periplasmic chaperone	1.05	6.25E-05	0.22	0.7261
yfcG	c2845	Hypothetical GST-like protein yccG	1.05	0.000316	0.19	NA
yhiO	c4292	Universal stress protein B	1.04	2.19E-09	0.32	0.5083
c4380	c4380	Conserved hypothetical protein	1.04	0.007464	0.19	NA
yhiV	c4325	Hypothetical protein yhiV	1.04	1.44E-08	0.00	0.9982
ygiW	c3763	Protein ygiW precursor	1.03	6.01E-13	0.21	0.6932
ygdI	c3380	Hypothetical lipoprotein ygdI precursor	1.03	5.32E-05	0.09	NA
c1437	c1437	Putative Rz endopeptidase from lambdoid prophage DLP12	1.02	0.008211	0.40	NA
dnaK	c0019	Chaperone protein dnaK	1.01	1.13E-09	0.75	0.1159
ccmH	c2731	Cytochrome c-type biogenesis protein ccmH precursor	-1.05	7.56E-09	-0.47	0.1233
tnaL	c5499	Tryptophanase leader peptide	-1.06	3.21E-14	-0.56	0.1599
fdnH	c1906	Formate dehydrogenase-N beta subunit	-1.11	0.001082	-0.35	0.4294
fdnI	c1907	Formate dehydrogenase-N gamma subunit	-1.32	9.01E-07	-0.46	0.3233
glnQ	c0894	Glutamine transport ATP-binding protein glnQ	-3.15	2.83E-45	0.84	0.0003
ybeJ	c0739	Glutamate/aspartate Periplasmic binding protein precursor	-3.20	8.73E-61	0.65	0.0596
pspA	c1774	Phage shock protein A	-3.64	1.88E-92	0.03	0.9693
glnP	c0895	Glutamine transport system permease protein glnP	-4.14	1.28E-54	0.77	0.0262
glnH	c0896	Glutamine-binding periplasmic protein precursor	-4.15	2.83E-144	0.36	0.2355
c0897	c0897	Hypothetical protein c0897	-4.56	4.04E-100	0.06	0.9306

Table S3

Differentially expressed genes in Δ*rpoN* EDL933 bacteria relative to wild-type EDL933 bacteria

Gene	R #	Product name	Log_2 fold change (Δ*rpoN* / wild-type)	Adjusted P-value	Log_2 fold change (Δ*rpoN* + pACYC-*rpoN* / wild-type)	Adjusted P-value
hisG	RS15120	ATP phosphoribosyltransferase	2.16	1.83E-57	0.38	0.0267

hisD	RS15125	histidinol dehydrogenase	1.87	2.87E-47	0.37	0.0266
hisH	RS15140	imidazole glycerol phosphate synthase subunit HisH	1.69	2.26E-21	0.51	0.0298
hisC	RS15130	histidinol-phosphate aminotransferase	1.67	1.71E-40	0.16	0.4368
tdcD	RS21295	propionate kinase	1.66	1.64E-05	1.62	0.0000
hisA	RS15145	1-(5-phosphoribosyl)-5-[(5-phosphoribosylamino) methylideneamino] imidazole-4-carboxamide isomerase	1.56	2.95E-21	0.43	0.0580
hisF	RS15150	imidazole glycerol phosphate synthase subunit HisF	1.56	1.58E-16	0.52	0.0121
tdcC	RS21300	threonine/serine transporter TdcC	1.48	0.000119	1.04	0.0355
hisB	RS15135	histidine biosynthesis bifunctional protein HisB	1.45	2.59E-33	0.18	0.2974
	RS15155	histidine biosynthesis bifunctional protein HisIE	1.33	1.87E-15	0.50	0.0203
glnL	RS25625	PAS domain-containing two-component system sensor histidine kinase	1.27	2.23E-28	0.41	0.0018
tdcE	RS21290	keto-acid formate acetyltransferase	1.22	0.014273	1.45	0.0000
glnG	RS25620	nitrogen regulation protein NR(I)	1.21	3.77E-24	0.52	0.0000
gadC	RS10510	glutamate/gamma-aminobutyrate antiporter	1.03	1.82E-16	-0.16	0.4466
	RS19100	formate hydrogenlyase subunit 5	1.02	0.000143	0.19	0.6983
sepD	RS24390	type III secretion system protein SepD	-1.00	0.014864	0.21	0.6682
queD	RS19320	6-carboxy-5,6,7,8-tetrahydropterin synthase	-1.02	7.05E-06	-0.88	0.0000
	RS27010	hypothetical protein	-1.02	7.22E-11	-0.94	0.0000
eae	RS24315	intimin	-1.03	6.60E-08	-0.06	0.8505
suhB	RS18125	inositol-1-monophosphatase	-1.03	2.25E-08	-0.69	0.0000
	RS12805		-1.04	0.009639	-1.15	0.0004
iprA	RS02170	transcriptional regulator	-1.05	0.004893	-1.01	0.0061
	RS21600		-1.06	0.030655	-1.16	0.0002

	RS16350	hypothetical protein	-1.08	0.005059	-1.23	0.0006
gltK	RS03545	glutamate ABC transporter permease	-1.09	6.97E-08	0.11	0.6757
	RS29215		-1.10	8.80E-06	-0.83	0.0003
ag43	RS05545		-1.10	0.323139	-1.25	0.2217
	RS12690	membrane protein	-1.11	0.000275	-0.94	0.0019
gltJ	RS03550	glutamate/aspartate ABC transporter permease GltJ	-1.12	3.22E-07	0.16	0.5527
	RS03620		-1.13	0.021777	-1.01	0.0000
escJ	RS24385	secretion system apparatus lipoprotein EscJ	-1.15	0.000577	0.00	0.9967
	RS25240		-1.19	0.035694	-1.37	0.0036
ag43	RS07695		-1.20	0.295889	-1.46	0.1506
	RS09430		-1.23	0.051114	-0.98	0.1249
	RS18960		-1.23	9.02E-11	-1.16	0.0000
	RS17255	hypothetical protein	-1.38	5.76E-10	-1.14	0.0030
	RS17500	hypothetical protein	-1.41	2.38E-07	-1.51	0.0000
	RS27810		-1.43	0.013834	-1.32	0.0084
cspH	RS06530	cold-shock protein CspH	-1.84	0.010752	-1.39	0.0550
glnQ	RS04640	glutamine ABC transporter ATP-binding protein	-2.68	1.38E-87	0.60	0.0000
gltI	RS03555	glutamate/aspartate ABC transporter substrate-binding protein	-2.97	2.76E-61	-0.16	0.5027
glnP	RS04645	glutamine ABC transporter permease	-2.98	1.66E-92	0.73	0.0000
pspA	RS11640	phage shock protein A	-3.27	4.14E-56	0.18	0.3970
glnH	RS04650	glutamine-binding periplasmic protein	-3.29	1.98E-217	-0.01	0.9755

References:

1. Hryckowian, A. J., Baisa, G. A., Schwartz, K. J., and Welch, R. A. (2015) *dsdA* Does Not Affect Colonization of the Murine Urinary Tract by *Escherichia coli* CFT073. *PLoS One* **10**, e0138121
2. Miranda, R. L., Conway, T., Leatham, M. P., Chang, D. E., Norris, W. E., Allen, J. H., Stevenson, S. J., Laux, D. C., and Cohen, P. S. (2004) Glycolytic and Gluconeogenic Growth of *Escherichia coli* O157:H7 (EDL933) and *E. coli* K-12 (MG1655) in the Mouse Intestine.*Infection and Immunity* **72**, 1666-1676
3. Schumacher, J., Behrends, V., Pan, Z., Brown, D. R., Heydenreich, F., Lewis, M. R., Bennett, M. H., Razzaghi, B., Komorowski, M., Barahona, M., Stumpf, M. P. H., Wigneshweraraj, S., Bundy, J. G., and Buck, M. (2013) Nitrogen and Carbon Status Are Integrated at the Transcriptional Level by the Nitrogen Regulator NtrC *In Vivo*. *mBio* 4:e00881–13.
